# Exploration of molecular mechanism of intraspecific cross-incompatibility in sweetpotato by transcriptome and metabolome analysis

**DOI:** 10.1007/s11103-022-01259-8

**Published:** 2022-03-25

**Authors:** Yiling Yang, Xiongjian Zhang, Hongda Zou, Jingyi Chen, Zhangying Wang, Zhongxia Luo, Zhufang Yao, Boping Fang, Lifei Huang

**Affiliations:** Guangdong Provincial Key Laboratory of Crop Genetic Improvement, Crops Research Institute, Guangdong Academy of Agricultural Sciences, Guangzhou, 510640 China

**Keywords:** Sweetpotato, Intraspecific cross-incompatibility, Molecular mechanism, Transcriptome, Metabolome

## Abstract

Cross-incompatibility, frequently happening in intraspecific varieties, has seriously restricted sweetpotato breeding. However, the mechanism of sweetpotato intraspecific cross-incompatibility (ICI) remains largely unexplored, especially for molecular mechanism. Treatment by inducible reagent developed by our lab provides a method to generate material for mechanism study, which could promote incompatible pollen germination and tube growth in the ICI group. Based on the differential phenotypes between treated and untreated samples, transcriptome and metabolome were employed to explore the molecular mechanism of sweetpotato ICI in this study, taking varieties ‘Guangshu 146’ and ‘Shangshu 19’, a typical incompatible combination, as materials. The results from transcriptome analysis showed oxidation–reduction, cell wall metabolism, plant–pathogen interaction, and plant hormone signal transduction were the essential pathways for sweetpotato ICI regulation. The differentially expressed genes (DEGs) enriched in these pathways were the important candidate genes to response ICI. Metabolome analysis showed that multiple differential metabolites (DMs) involved oxidation–reduction were identified. The most significant DM identified in comparison between compatible and incompatible samples was vitexin-2-O-glucoside, a flavonoid metabolite. Corresponding to it, *cytochrome P450s* were the most DEGs identified in oxidation–reduction, which were implicated in flavonoid biosynthesis. It further suggested oxidation–reduction play an important role in sweetpotato ICI regulation. To validate function of oxidation–reduction, reactive oxygen species (ROS) was detected in compatible and incompatible samples. The green fluorescence was observed in incompatible but not in compatible samples. It indicated ROS regulated by oxidation–reduction is important pathway to response sweetpotato ICI. The results in this study would provide valuable insights into molecular mechanisms for sweetpotato ICI.

**Supplementary Information:**

The online version contains supplementary material available at 10.1007/s11103-022-01259-8.

## Introduction

Sweetpotato (*Ipomoea batatas* (L.) Lam.), a hexaploid plant, is an economically important root crop that is widely used as a food, feed, and fuel resource worldwide(Wei et al. 2019; Zhang et al. 2020a; Fan et al. 2021). It is the sixth most important crop after rice, maize, wheat, potato, and cassava in the word (Drapal et al. 2019) and considered as a healthy food for human due to its rich source of nutritional components, such as starch, dietary fiber, flavonoids, phenolics, vitamins, carotenoids, and other nutrients (Hou et al. 2019; Zhang et al. 2020b). Cross-breeding is the major method for sweetpotato breeding. However, cross-incompatibility, occurring frequently in intraspecific hybridization, seriously affects the breeding and germplasm resource utilization of sweetpotato, especially for the varieties with excellent agronomic traits, which greatly hinders their usage in breeding. Therefore, the mechanism study of intraspecific cross-incompatibility (ICI) is of great significance for sweetpotato breeding.

Because the similar phenotypes between ICI and self-incompatibility (SI) in sweetpotato, ICI varieties are usually supposed to be the same S-haplotype of SI system. The common performances are little pollen stucking on the stigma, no pollen germination on the stigma, and intense callose reaction in papillae(Ketong and Shuyun 1992; Shuyun and Taiyuan 1992). SI has been extensively studied in plant, including sporophytic SI (SSI) and gametophytic SI (GSI) (Duan et al. 2020). SSI has been clearly elucidated in *Brassicaceae*, with SP11/SCR (S-locus protein 11 or S-locus cysteine-rich protein) located at pollen coats as the male determinant and SRK (S-locus receptor kinase) localized in the stigmatic papilla cells as the female determinant (Bedinger et al. 2017; Dou et al. 2021). GSI is mainly studied in *Solanaceae*, *Rosaceae* and *Rutaceae* (Kong et al. 2021), including petunia (Zhao et al. 2021), potato (Enciso-Rodriguez et al. 2019), pear (Chen et al. 2018), citrus (Liang et al. 2020), and so on. Especially for potato, except female determinant S-RNase (Ye et al. 2018), a new *S-locusinhibitor* (*Sli*) gene was identified, which could break SI in diploid potatoes by inhibiting S-RNase (Ma et al. 2021). Based on these, genome design of hybrid potato was accomplished in 2021 (Zhang et al. 2021a).

Sweetpotato is considered to be SSI system, according to the phenotype of pollen on stigma and studies in diploid *Ipomoea trifida* (Rahman et al. 2007a, b). However, it has been reported to be different from SSI in *Brassicaceae* (Fujii and Takayama 2017; Koseva et al. 2017)and the mechanism remains unclearly. The study of sweetpotato ICI will be helpful to uncover the mystery of sweetpotato SI and provide an effective mean to breakdown them. Nevertheless, hexaploidy and complicated genetic background make it more difficult to explore the mechanism of SI and ICI in sweetpotato. Fortunately, our lab has screened an inducible reagent after years of research, which can weaken the sweetpotato ICI and promote the pollen germination and seed setting in sweetpotato ICI combinations (Zhang et al. 1998). Application of the inducible reagent in ICI combinations can provide valuable materials for the mechanism study of ICI. Based on these facts, we carried out transcriptome and metabolome analysis to compare the difference of genes and metabolites between treated and untreated pollination stigmas in this study to explore the molecular mechanism of sweetpotato ICI. The results suggested that oxidation–reduction, cell wall metabolism, plant hormone signal transduction and plant–pathogen interaction were the important pathways for ICI regulation. This study provides an valuable insight into molecular mechanisms of sweetpotato ICI and is significant for further research of gene function.

## Materials and methods

### Plant materials and treatments

The sweetpotato varieties, including ‘Guangshu 146’, ‘Guangshu 79’, and ‘Shangshu 19’, were used in this study, which were grown in the National Germplasm Guangzhou Sweetpotato Nursery, China. ‘Guangshu 146’ and ‘Guangshu 79’ are the carotenoid-rich varieties selected by Crops Research Institute, Guangdong Academy of Agricultural Sciences. ‘Shangshu 19’ is a high-starch variety bred by Shangqiu Academy of Agricultural and Forestry Sciences. ‘Guangshu 146’ and ‘Shangshu 19’ are the cross-incompatibility group, while ‘Guangshu 146’ and ‘Guangshu 79’ are the cross-compatibility group. Like most sweetpotato varieties, ‘Guangshu 146’, ‘Guangshu 79’ and ‘Shangshu 19’ have few flowers in field. In order to promote flowering for the three varieties, *Ipomoea carnea* was used as rootstock for grafting. The grafting seedlings were cultivated in plastic pots in September in Guangzhou, China, with culture media composed of peat soil (Jiffy product), field soil and sand (volume ratio 3:5:2), and maintained in greenhouse.

The grafting seedlings flowered after one month cultivation, and were used in the experiments. The floral buds were tied with string 1 day prior to anthesis to avoid the pollution of foreign pollens. Pollination was carried out during 8:00–12:00 a.m. on next day. The pollens of ‘Guangshu 79’ were normally pollinated on ‘Guangshu 146’ to serve as control for the incompatibility group of ‘Guangshu 146’ and ‘Shangshu 19’. Stigmas of ‘Guangshu 146’ and ‘Shangshu 19’ were pollinated by each other pollens with or without treatment of inducible reagent as previously described (Zhang et al. 1998). After 4 h, the treated and untreated pollinated stigmas were collected to determine pollen germination to identity the compatibility of different cross groups, with three biological replicates (3 to 5 pollinated stigmas for each replicate) for each sample. Additionally, to select the sampling time for transcriptome and metabolome analysis, the pollinated stigmas of ‘Guangshu 146’ × ‘Guangshu 79’ (untreated) and ‘Guangshu 146’ × ‘Shangshu 19’ (treated) were also collected to determine the pollen germination at 5, 10, 20, 30, 60, and 120 min after pollination, with three biological replicates (5 to 10 pollinated stigmas for each replicate) for each time point. The normal (CK), inducible reagent treated (MT), pollinated by ‘Shangshu 19’ pollens (FT), and pollinated by ‘Shangshu 19’ pollens after inducible reagent treated (MFT) stigmas of ‘Guangshu 146’ were used as transcriptome materials due to their larger size. The stigmas of Guangshu 146 pollinated by ‘Guangshu 79’ pollens (FT-G79), CK, FT, and MFT were used as metabolome materials. All samples were collected at 1 h after treatment, immediately frozen in liquid nitrogen, and maintained at − 80 °C, with three biological replicates for each sample.

### Determination of pollen germination

The fresh samples of pollinated stigmas with or without treatment of inducible reagent were put on glass slides and covered with dye reagent (1:1:1:1.5 mixture of phenol/glycerinum/lactic acid/distilled water, eosin stain, dissolved by heating). The glass slides were evenly heated on alcohol lamp until the dye reagent was boiled, and then covered with coverslips on the samples. The prepared glass slides were used to observe the pollen germination by OLYMPUS CKX41 microscope.

### Transcriptome analysis

#### RNA isolation, cDNA library construction and sequencing

Total RNA was isolated from FT, MT, MFT, and CK samples using RNAprep Pure Plant Plus Kit (Tiangen, Beijing, China), with three biological replicates for each sample. Degradation of RNA were assessed on 1% agarose gels by two distinct bands appearing without dispersion. Purity and concentration of RNA were determined by NanoDrop 2000 (Thermo Scientific, DE, USA). The purity was evaluated by the ratio of OD_260/280_, with 2.0 being the best, but not lower than 1.8. RNA integrity was examined using Agilent 2100 Bioanalyzer (Agilent Technologies, Palo Alto, California) through RIN (RNA integrity number), with 10 being best, but more than 6.3 is enough for plant. The purified RNA samples were employed to construct cDNA libraries using NEBNext® Ultra™ RNA Library Prep Kit for Illumina® (NEB, USA) according to the manufacturer’s protocols. The Agilent Bioanalyzer 2100 system was used to evaluate the constructed libraries, and the Illumina HiSeq™ 2000 platform was used for sequencing.

#### Data analysis

Reads containing adapter or ploy-N and low-quality reads were eliminated from the raw data. TopHat v2.0.12 was used to align the paired-end clean reads to the sweetpotato genome (http://public-genomes-ngs.molgen.mpg.de/SweetPotato/). The read numbers mapped to each gene were calculated using HTSeq v0.6.1. FPKM (fragments per kilo base of transcript per million mapped reads) was employed to determine the gene expression level according to the gene length and read count mapped to this gene. Differential expression analysis of FT vs. CK, MFT vs. CK, MFT vs. MT and MFT vs. FT comparisons were carried out using the DESeq R package (1.18.0). Genes with an adjusted P-value < 0.05 identified by DESeq were considered as differentially expressed genes (DEGs). All clean and processed transcriptomic sequence data used in this research were deposited in the Sequence Read Archive (SRA) under the accession number PRJNA611841. DEGs of different comparisons were further analyzed by gene ontology (GO) and Kyoto encyclopedia of genes and genomes (KEGG) enrichment. GO and KEGG enrichment analyses were conducted using the GOseq R package and KOBAS software. GO terms and KEGG pathways with corrected P value less than 0.05 were regarded to be remarkably enriched by DEGs.

### Quantitative real-time PCR (RT-qPCR)

Total RNA was extracted as previously described. Subsequently, 1 µg RNA was reversely transcribed into cDNA using FastKing gDNA Dispelling RT SuperMix (Tiangen, Beijing, China). The specific primers were designed using NCBI primer blast (Table S3). The primers' specificity and efficiency was evaluated by semi-quantitative RT-PCR and RT-qPCR through 1% agarose gels and peak figure in one or two cDNA samples. RT-qPCR was performed on a CFX96TM Real-time PCR System (Bio-Rad, USA) using ChamQTM Universal SYBR® qPCR Master Mix (Vazyme, China), and data was analyzed by CFX detection system software (version 3.1). Each experiment was performed in triplicate. The relative expressions of target genes were calculated using the 2^−ΔΔCt^ method. According to Zhang et al. (2019), the sweetpotato actin gene was utilized as the internal control. The expression of genes in MT, FT and MFT was normalized by CK.

### Metabolome analysis

The freeze-dried samples of CK, FT, FT-G79, and MFT were crushed for 1.5 min at 30 Hz using a mixer mill (MM 400, Retsch) with a zirconia bead. Moreover, 100 mg powder was weighted and extracted overnight at 4℃ with 0.6 mL 70% aqueous methanol. The extracts were centrifuged for 10 min at 10,000 × g, and subsequently absorbed (CNWBOND Carbon-GCB SPE Cartridge, 250 mg, 3 mL; ANPEL, Shanghai, China, www.anpel.com.cn/cnw) and filtrated (SCAA-104, 0.22-μm pore size; ANPEL, Shanghai, China, http://www.anpel.com.cn/) before UPLC–MS/MS analysis. The extracts were analyzed using an UPLC–ESI–MS/MS system (UPLC, Shim-pack UFLC SHIMADZU CBM30A system, www.shimadzu.com.cn/; MS, Applied Biosystems 4500 Q TRAP, www.appliedbiosystems.com.cn/). The analytical conditions were as described previously (Chen et al. 2013). Identification and quantification of metabolites were based on MWDB database (MetWare Biological Science and Technology Co., Ltd., Wuhan, China) and multiple reaction monitoring mode (Fraga et al. 2010). Metabolite data was analyzed by the Analyst 1.6.3 software (AB SCIEX, 86 Ontario, Canada). Principal component analysis (PCA) of CK, FT, FT-G79 and MFT samples was performed to observe differences in metabolic composition by R (www.r-project.org). Hierarchical cluster analysis (HCA) was carried out by R package pheatmap, and the results of samples and metabolites were presented as heatmaps with dendrograms. Metabolites with variable importance in projection (VIP) ≥ 1 and absolute log_2_FC (fold change) ≥ 1 were identified as differential metabolites (DMs) for group discrimination.

### Reactive oxygen species (ROS) detection

ROS detection was carried out in the stigmas of ‘Guangshu 146’ pollinated by self-pollens (SP) and stigmas of CK, FT, FT-G79 and MFT, which was performed three times at different time points, with three biological replicates for each sample in each time. Protocol for stigmatic ROS detection followed that of *Brassica rapa* (Zhang et al. 2021b). 2′,7′-Dichlorodihydrofluorescein diacetate (H2DCFDA) was used as probe to detect ROS. After 1 h pollination, the stigmas were soaked in MES–KCl buffer (MES 10 mM, KCl 5 mM, CaCl_2_ 50 mM, pH 6.15) for 30 min, subsequently stained with 50 μM H2DCFDA for 1 h, and washed at least 3 times before observation. ROS in stigma was observed with Zeiss 710 laser scanning confocal microscope.

### Statistical analysis

In this study, the means and standard errors of the data were calculated using Microsoft Excel formulas. Analysis of variance (ANOVA) was used to detect differences among samples by SPSS 19.0 (SPSS, Inc., Chicago, IL, USA), and the least significant difference (LSD) was chosen to compare the means at the P < 0.05 level.

## Results

### Treatment of inducible reagent promotes the pollen germination in sweetpotato ICI combination

**Fig. 1:**
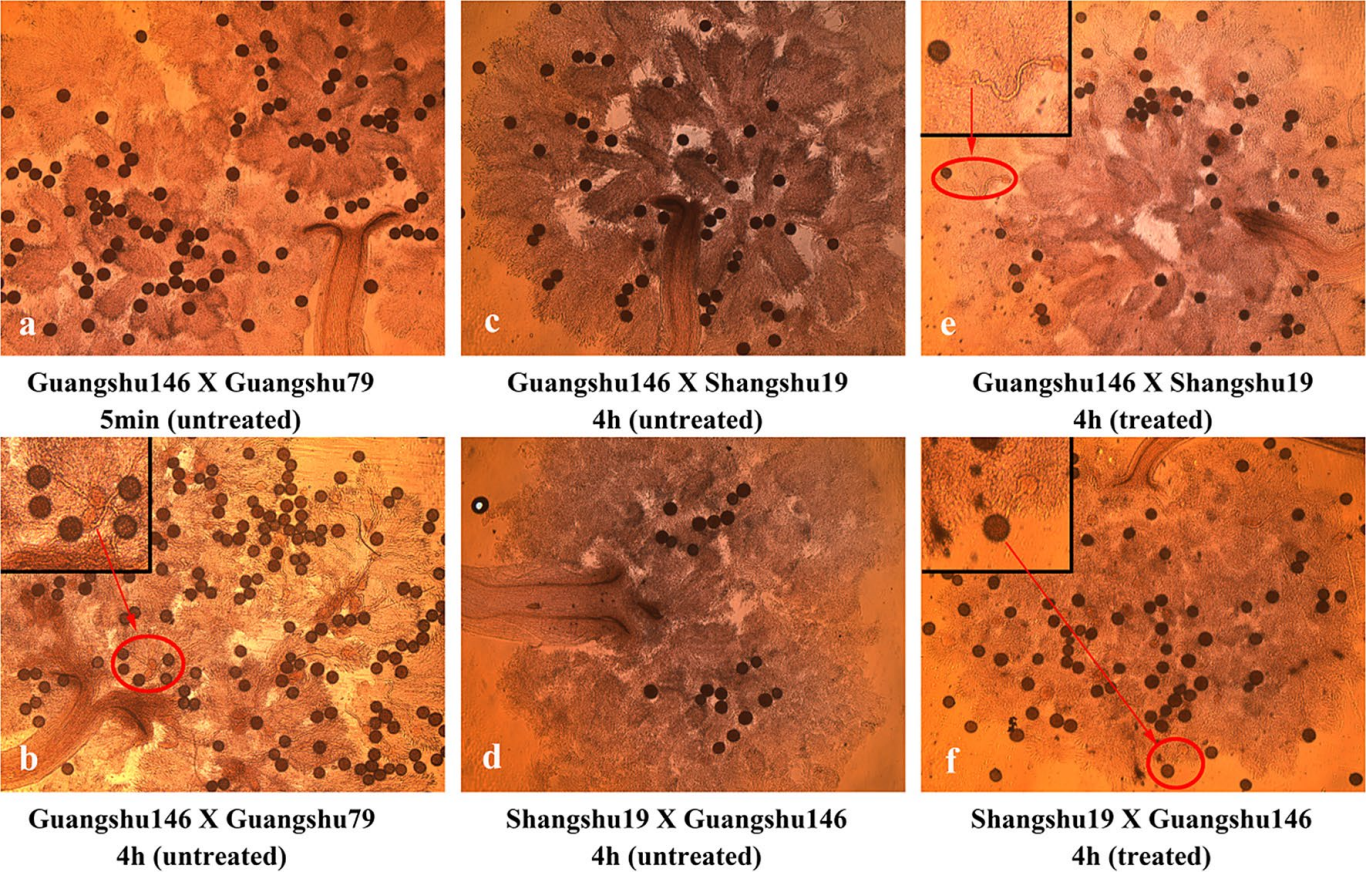
Microscopic observation of pollen germination under treated and untreated conditions by inducible reagent. **a**, **b** Pollens of compatibility group ‘Guangshu 146 × Guangshu 79’ after 5 min and 4 h pollination under the untreated condition. **c**, **d** Pollens of incompatibility groups ‘Guangshu 146 × Shangshu 19’ and ‘Shangshu 19 × Guangshu 146’ after 4 h pollination under the untreated condition. **e**, **f** Pollens of incompatibility groups ‘Guangshu 146 × Shangshu 19’ and ‘Shangshu 19 × Guangshu 146’ after 4 h pollination under the treated condition. The black boxes are the enlarged images of germinated pollens pointed by red rows

According to “Descriptors and Data Standard for Sweetpotato”, the compatibility between sweetpotato varieties is judged by pollen germination after 4 h pollination (Zhang and Fang 2006). In this study, pollen germination was firstly observed in a compatibility combination between ‘Guangshu 146’ and ‘Guangshu 79’. Most of the pollens germinated after 4 h of pollination, while they were not observed after 5 min pollination (Fig. 1a, b). Taking them as control, the pollen germination was detected in a reciprocal cross between ‘Guangshu 146’ and ‘Shangshu 19’. No pollen was detected to germinate after 4 h pollination (Fig. 1c, d), indicating that ‘Guangshu 146’ and ‘Shangshu 19’ were the typical ICI combination. However, when the stigmas were treated by inducible reagent before pollination, pollen germination was detected in the reciprocal cross after 4 h pollination (Fig. 1e, f). It supported that the inducible reagent could promote sweetpotato ICI breaking and generate materials for mechanism research.

**Fig. 2:**
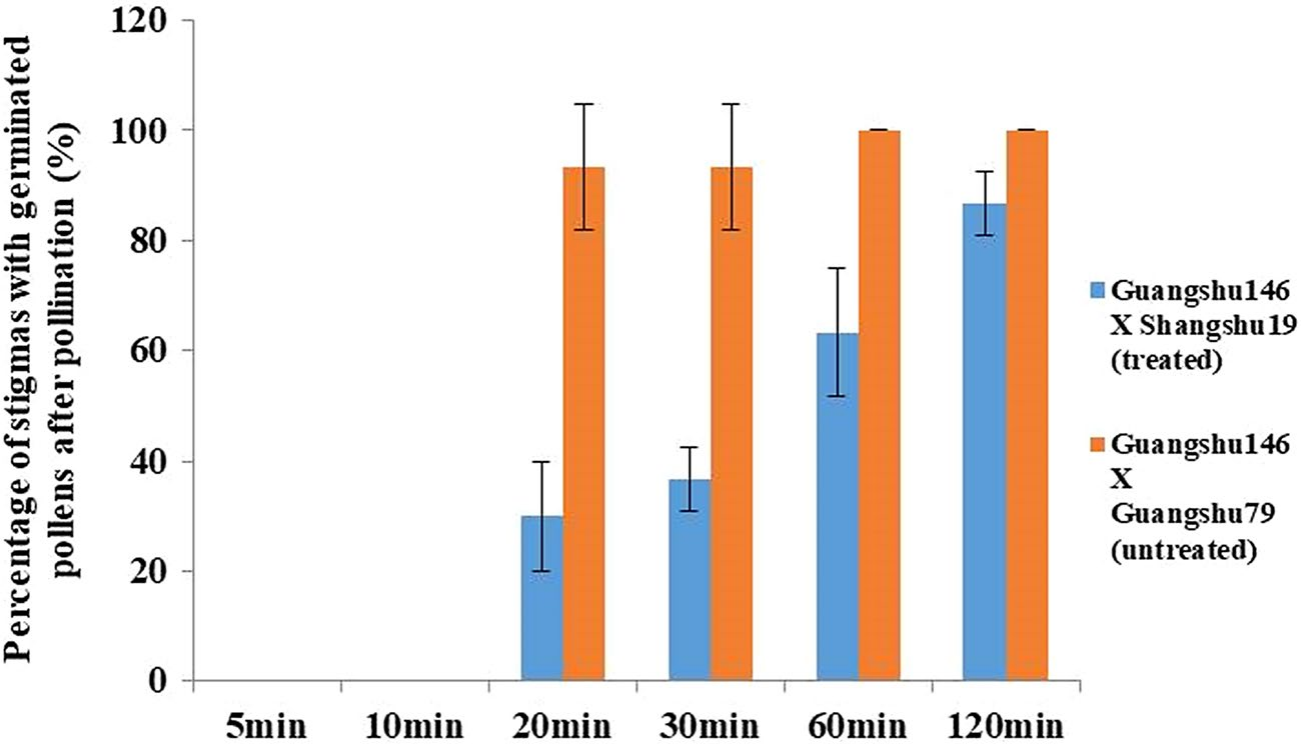
Statistics of stigmas with germinated pollens at different time points after pollination of ‘Guangshu 146 × Shangshu 19’ (treated) and ‘Guangshu 146 × Guangshu 79’ (untreated). Error bars represent the SD of triplicate assays

To select appropriate sampling time for transcriptome and metabolome analysis, pollen germination was also detected at 5, 10, 20, 30, 60, and 120 min after pollination in combination ‘Guangshu 146’ × ‘Shangshu 19’ (treated by inducible reagent), taking ‘Guangshu 146’ × ‘Guangshu 79’ as control. The results showed that pollen germination began at 20 min after pollination in treated and control samples, while it did not happen after 5 and 10 min. Even so, only 30% and 36.67% stigmas were detected pollen germination at 20 and 30 min after pollination in treated samples. However, it became 63.33% and 86.67% at 60 and 120 min, respectively (Fig. 2). Compared with 120 min when the pollen germination occurred in most of the pollinated stigmas in treated samples, more pollinated pollens were ready or beginning to germinate at 60 min. Therefore, it suggested that 60 min was the best time point for transcriptome and metabolome sampling.

### RNA-Seq and analysis of DEGs

A total of 637.406 million raw reads were created by RNA-Seq in CK, MT, FT and MFT samples. After adapter- or ploy-N-containing and low-quality reads filtered, 610.157 million clean reads were obtained (Q20 > 96%). After mapping them to the sweetpotato genome, 462.351 million reads were obtained, including 118.802, 118.424, 117.920, and 107.204 million reads in CK, FT, MT, and MFT samples, respectively. The mapped reads of the four samples accounted for 76.04%, 76.01%, 75.91%, and 75.07% of each total clean reads, respectively, and the uniquely mapped reads were 71.33%, 71.22%, 71.25%, and 70.47% (Table S1), respectively. The mapped reads were assembled using Cufflinks, followed by a comparison with the known gene model using Cuffcompare, a total of 62,739 genes were obtained, and 7287 novel genes were identified.

**Fig. 3:**
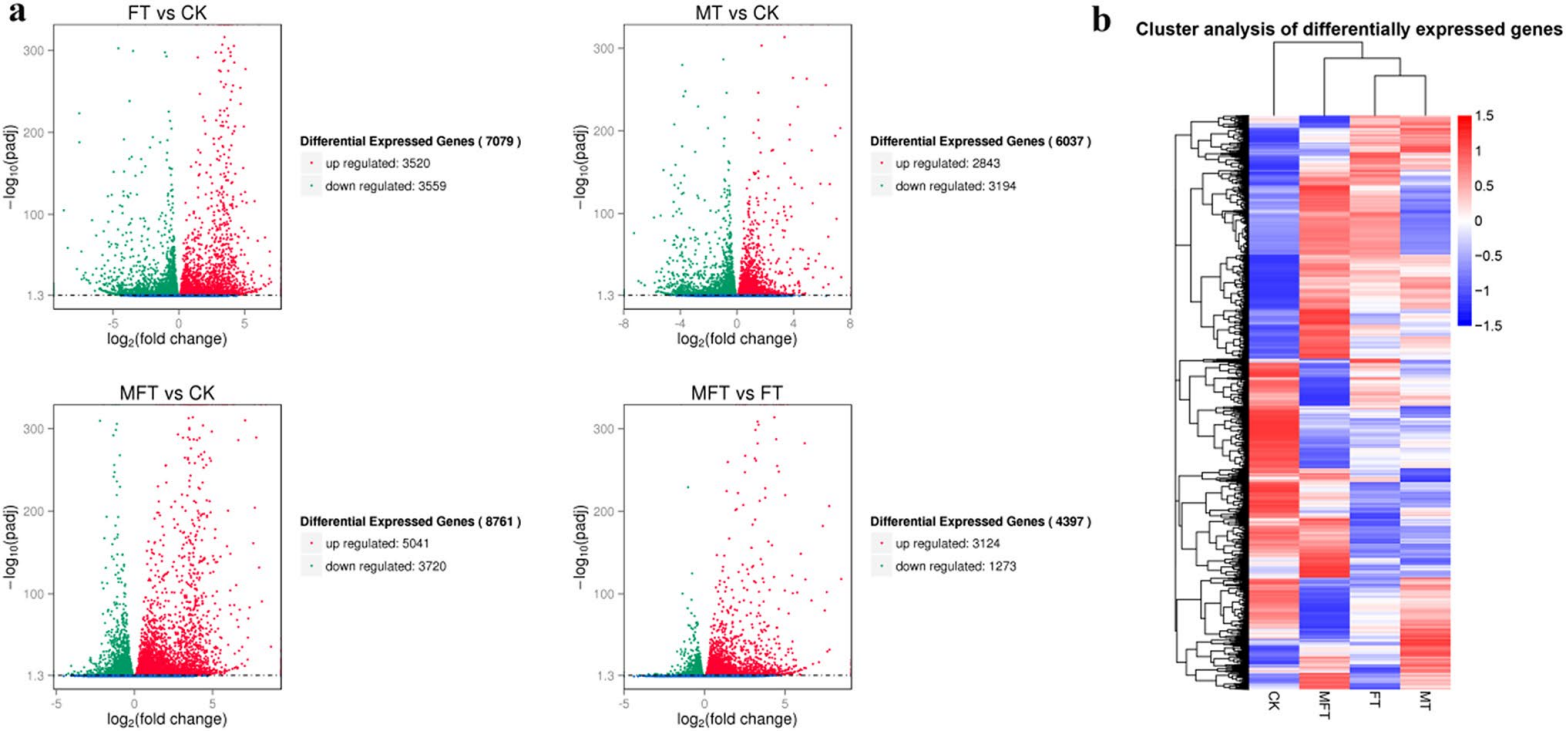
Analysis of differentially expressed genes (DEGs) in different comparison groups. **a** Number of DEGs in FT vs. CK, MT vs. CK, MFT vs. CK and MFT vs. FT. **b** Cluster analysis of DEGs

DEG analysis showed that there were 7079, 6037, 8761, and 4397 DEGs detected in comparisons of FT vs. CK, MT vs. CK, MFT vs. CK, and MFT vs. FT, including 3520 and 2843 up-regulated, and 3559 and 3194 down-regulated DEGs in comparisons of FT vs. CK and MT vs. CK, respectively, but 5401 and 3124 up-regulated, and 3720 and 1273 down-regulated DEGs in comparisons of MFT vs. CK and MFT vs. FT (Fig. 3a), respectively. It indicated that the inducible reagent induced more DEGs in pollinated stigmas, and more up-regulated DEGs in comparisons of MFT vs. CK and MFT vs. FT might promote ICI breaking in MFT sample. Cluster analysis based on DEGs showed that FT and MT samples were clustered into one group but different from the MFT (Fig. 3b). It implied that the response of stigma to incompatible pollens was similar to sole treatment by inducible reagent, but knowing from MFT. Therefore, MT could be the other control sample like FT, which could be used for MFT analysis.

### GO functional annotation and identification of DEGs response to sweetpotato ICI

GO enrichment analysis of DEGs showed that ‘oxidoreductase activity’ and ‘oxidation–reduction process’ were the most significant GO terms in comparisons of FT vs. CK and MT vs. CK, and ‘cell wall organization or biogenesis’ and ‘polygalacturonase activity’ were the significant GO terms in the comparison of MFT vs. CK. All of them were also the significantly enriched GO terms in the comparison of MFT vs. FT (Fig. S1). It suggested that oxidation–reduction and cell wall metabolism were the important pathways for sweetpotato ICI response.

#### Response of oxidation–reduction to sweetpotato ICI

**Fig. 4:**
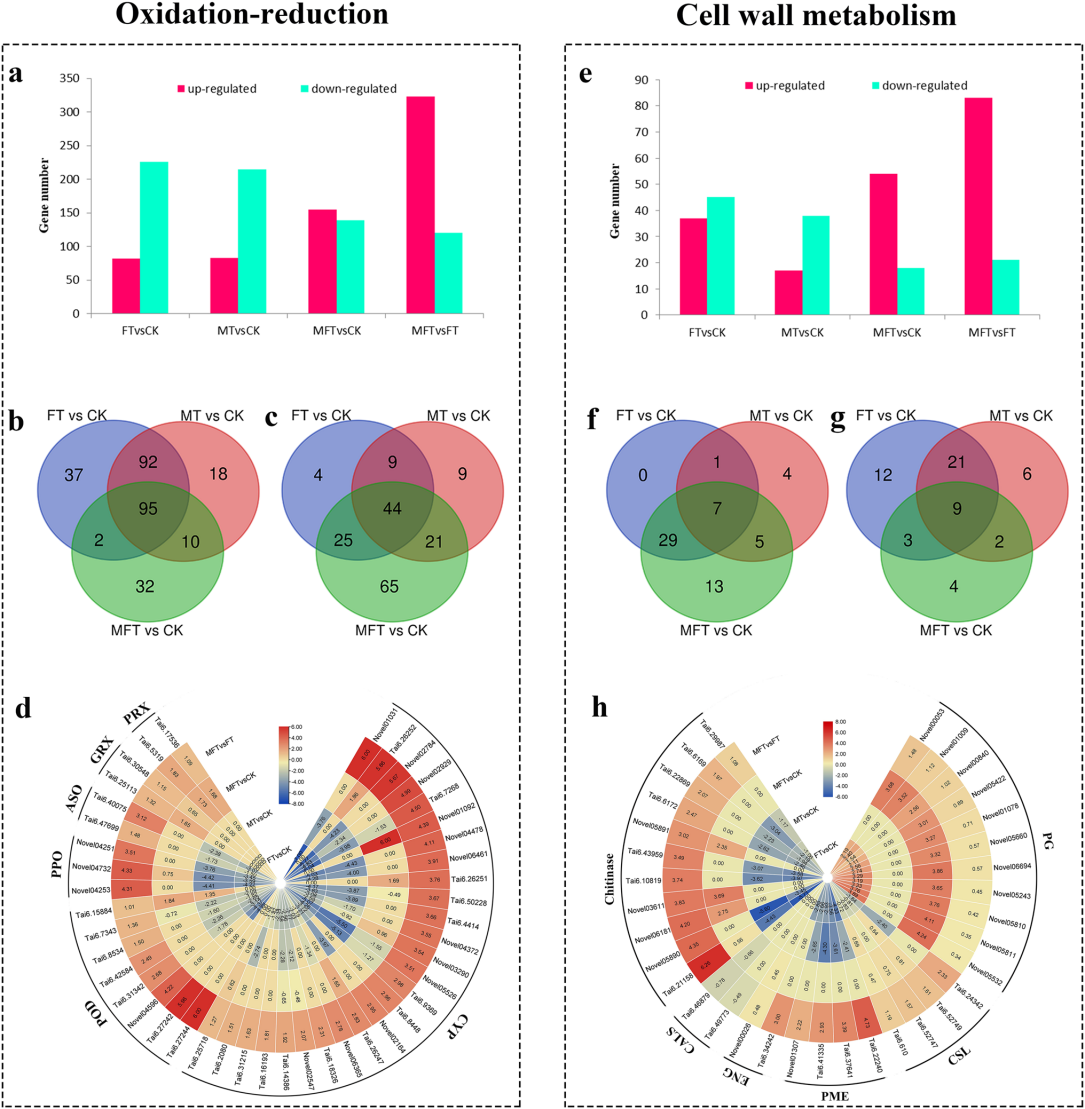
Analysis of DEGs enriched in oxidation–reduction and cell wall metabolism GO terms in MFT vs. FT. **a** The number of DEG from MFT vs. FT and oxidation–reduction GO terms in different comparison groups. **b**, **c** Venn diagram for down-regulated and up-regulated DEGs enriched in oxidation–reduction GO terms respectively. **d** Heatmap of selected DEGs from oxidation–reduction GO terms. **e** The number of DEG from MFT vs. FT and cell wall metabolism GO terms in different comparison groups. **f**, **g** Venn diagram for down-regulated and up-regulated DEGs enriched in cell wall metabolism GO terms respectively. **h** Heatmap of selected DEGs from cell wall metabolism GO terms

After collection of all DEGs enriched in the GO terms involved in oxidation–reduction, 443 DEGs were obtained from MFT vs. FT, including 323 up- and 120 down-regulated genes, respectively, 308, 298, and 294 DEGs of them were differentially expressed in comparisons of FT vs. CK, MT vs. CK, and MFT vs. CK, respectively (Fig. 4a). Expression analysis showed that there were 82 and 83 up-regulated, and 226 and 215 down-regulated genes in comparisons of FT vs. CK and MT vs. CK, but 155 up- and 139 down-regulated genes in the comparison of MFT vs. CK, respectively. Among them, 129 DEGs were down-regulated in FT vs. CK but not MFT vs. CK, 92 of them were shared DEGs between FT vs. CK and MT vs. CK, while only 32 DEGs were especially down-regulated in MFT vs. CK (Fig. 4b). On the contrary, there were 65 DEGs especially up-regulated in MFT vs. CK, but only 22 especially in FT vs. CK and MT vs. CK (Fig. 4c). It implied that oxidation-reduction was important for sweetpotato ICI regulation, and down-regulation of genes in oxidation–reduction might be the reason for the incompatibility in FT.

Function analysis of the DEGs enriched in oxidation–reduction revealed that 48 DEGs were identified as *cytochrome P450* (*CYP*) in MFT vs. FT, 35 of them were up-regulated, and 26 of them were log_2_ > 1. In the 26 DEGs, most of them were down-regulated in FT vs. CK and MT vs. CK, but no differentially expressed or up-regulated in MFT vs. CK (Fig. 4d). It declared that *CYP* might participate in oxidation–reduction regulation in ICI reponse. In addition, 11 DEGs were identified as *peroxidase* (*POD*) in MFT vs. FT, 10 of them were up-regulated, and eight of them were log_2_ > 1. Similar to the *CYP*, most DEGs with log_2_ > 1 were down-regulated in FT vs. CK and MT vs. CK, but no differentially expressed in MFT vs. CK. Besides, several *polyphenol oxidase* (*PPO*) and *L-ascorbate oxidase* (*ASO*) genes were also identified, with similar expression pattern to *POD* (Fig. 4d). It suggested that the down-regulation of oxidation–reduction enzyme genes was the factor to result in incompatibility in FT. Meanwhile, several DEGs were identified as *peroxiredoxin* (*PRX*) and *glutaredoxin* (*GRX*). All of them were up-regulated in MFT vs. FT and MFT vs. CK, but no differentially expressed in FT vs. CK and MT vs. CK (Fig. 4d), indicating that the up-regulation of oxidation–reduction protein gene was helpful for the incompatibility breaking in MFT.

#### Response of cell wall metabolism to sweetpotato ICI

After the collection of all DEGs enriched in the GO terms involved in cell wall metabolism, 104 DEGs were obtained from MFT vs. FT, including 83 up- and 21 down-regulated genes, 82, 55, and 72 DEGs of them were differently expressed in FT vs. CK, MT vs. CK and MFT vs. CK respectively (Fig. 4e). Among them, a total of 59 DEGs were up-regulated in the three combinations, 54 DEGs were up-regulated in MFT vs. CK, 18 of them were DEGs of MFT vs. CK, but not FT vs. CK, while only 1 DEG was up-regulated in FT vs. CK, but not MFT vs. CK (Fig. 4f). On the contrary, a total of 57 DEGs were down-regulated in the three combinations, 45 DEGs were down-regulated in FT vs. CK, 33 of them were DEGs in FT vs. CK, but not MFT vs. CK, including 21 shared DEGs between FT vs. CK and MT vs. CK, while only 4 DEGs were specially down-regulated in MFT vs. CK (Fig. 4g). It suggested that down-regulation of genes in cell wall metabolism resulted in incompatibility in FT, while genes up-regulation promoted the incompatibility breaking in MFT.

Function analysis of DEGs from MFT vs. FT showed that numerous DEGs were identified as *Endo-1,3(4)-beta-glucanase (ENG)*, *pectinesterase (PME)*, *cellulose synthase-like protein (CSL)*, *Callose synthase (CALS)*, and *polygalacturonase (PG)* (Fig. 4h). They were genes of important enzymes for cell wall metabolism. Among them, 21 DEGs were identified as *PGs*, 18 of them were up-regulated in MFT vs. FT, 11 of them were up-regulated in FT vs. CK and MFT vs. CK, but not differentially expressed in MT vs. CK, which indicated that *PGs* might function in pollen. Unlike *PGs*, four DEGs were identified as *CSLs*, and all performed log_2_ > 1 in MFT vs. FT, which were down-regulated in FT vs. CK, but up-regulated or not differentially expressed in MFT vs. CK. In addition, 16 DEGs were identified as *PMEs*, and 13 of them were up-regulated in MFT vs. FT, with 4 DEGs being log_2_ > 1. The four *PMEs* were down-regulated in FT vs. CK and MT vs. CK, but not differentially expressed in MFT vs. CK. Moreover, two *ENGs* were also identified and up-regulated in MFT vs. FT, while two *CALSs* were down-regulated in MFT vs. FT. It indicated that *ENG*, *PME*, *CSL*, *CALSB*, *PG* might be implicated in incompatibility regulation in FT and MFT. Interestingly, in MFT vs. FT, 13 DEGs were identified as *chitinases* (*CHI*), and all of them were up-regulated. Moreover, 11 of them were log_2_ > 1, which were mostly down-regulated in FT vs. CK and MT vs. CK, but not differentially expressed or up-regulated in MFT vs. CK (Fig. 4h). It speculated that *CHI* took part in incompatibility regulation.

#### Identification of DEGs involved in pollen–pistil interaction

Furthermore, pollen–pistil interaction was also the enrichment GO term in MFT vs. FT. Attractively, eight DEGs enriched in the GO term were all identified as *receptor-like serine/threonine-protein kinase*s (*RLKs*) (Table 1). Five of them were log_2_ > 1 and identified as *G-type RLKs* in MFT vs. FT, which were all down-regulated in FT vs. CK but not expressed in MFT vs. CK. It indicated that the *G-types RLKs* might play important roles in signal transduction for sweetpotato ICI regulation, and the down-regulation of them might promote ICI in the FT sample.

**Table 1:** Differentially expressed genes (DEGs) enriched in pollen–pistil interaction GO term in MFT vs. FT

Gene ID	Log_2_ fold change				Blast Swiss Prot
	FT vs. CK	MT vs. CK	MFT vs. CK	MFT vs. FT	
Tai6.12922	− 4.8420			4.6359	G-type lectin S-receptor-like serine/threonine-protein kinase
Tai6.4028	− 4.4793			4.4764	G-type lectin S-receptor-like serine/threonine-protein kinase
Tai6.31339	− 2.6006	− 1.6087		2.7172	G-type lectin S-receptor-like serine/threonine-protein kinase
Tai6.20133	− 2.0689	− 1.5175		2.5682	G-type lectin S-receptor-like serine/threonine-protein kinase
Tai6.22155	− 1.3349			1.2124	G-type lectin S-receptor-like serine/threonine-protein kinase
Tai6.6202			0.3289	0.2401	Receptor-like serine/threonine-protein kinase SD1-7
Tai6.5403	− 0.3016	− 0.3274	− 0.4112	− 0.1172	Receptor-like serine/threonine-protein kinase SD1-8
Novel00452			− 2.1661	− 2.1012	G-type lectin S-receptor-like serine/threonine-protein kinase

### KEGG enrichment analysis and identification of DEGs involved in sweetpotato ICI regulation

#### Implication of plant–pathogen interaction in sweetpotato ICI regulation

**Fig. 5:**
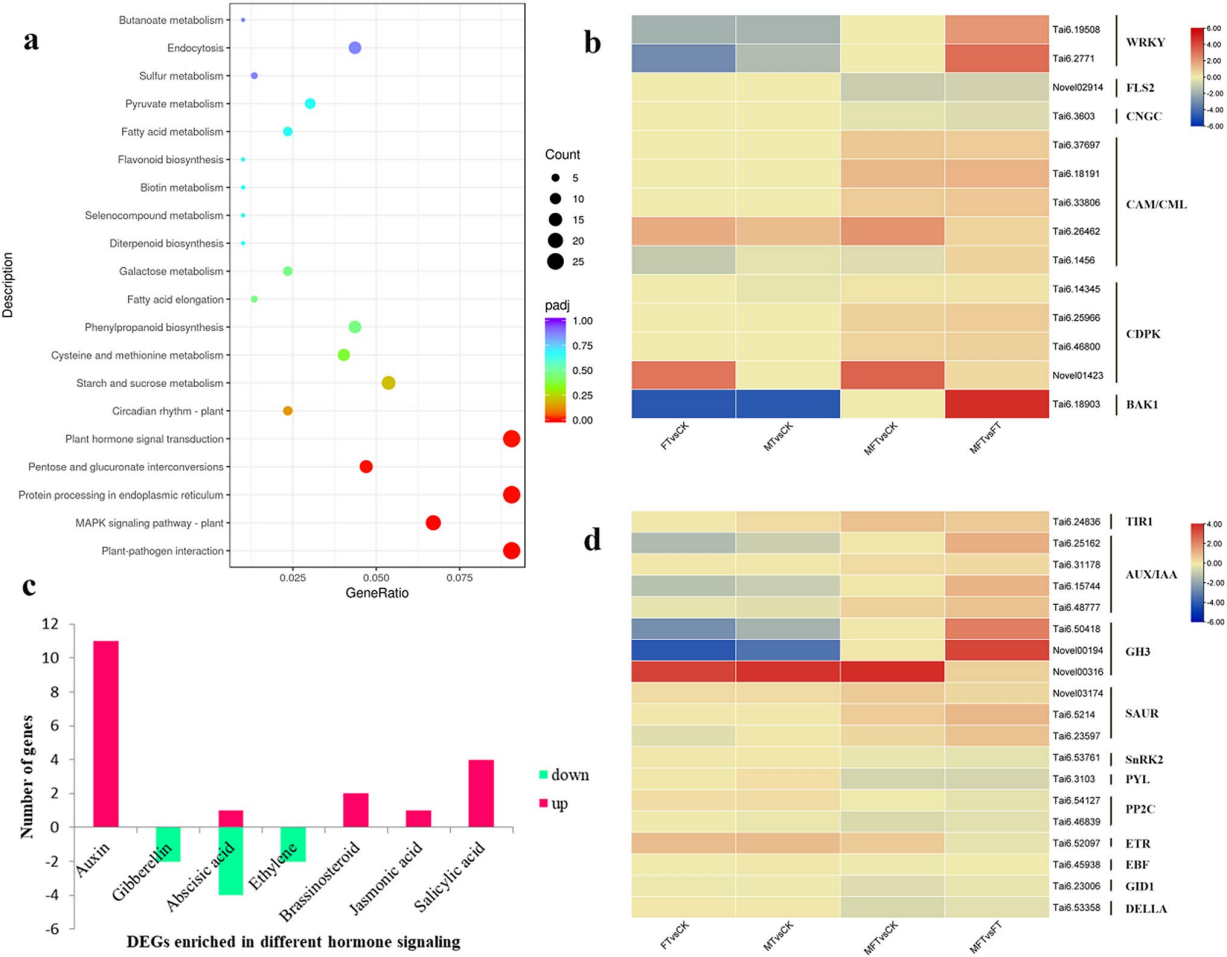
Analysis of DEGs enriched in plant–pathogen interaction and plant hormone signal transduction pathways from KEGG enrichment analysis in MFT vs. FT. **a** The scatter diagram of DEGs enriched in KEGG pathways in MFT vs. FT. **b** Heatmap of the identified DEGs enriched in plant–pathogen interaction pathway. **c** The number of DEG enriched in different hormone signaling in MFT vs. FT. **d** Heatmap of the identified DEGs enriched in plant hormone signal transduction pathway

KEGG analysis showed that plant–pathogen interaction, MAPK signaling pathway-plant, pentose and glucuronate interconversions, and plant hormone signal transduction were the significant enrichment pathways in MFT vs. FT (Fig. 5a). Plant–pathogen interaction was the most significant pathway, which was attributed to the similarity between pollen–pistil and plant–pathogen interaction. Moreover, 27 DEGs were enriched in the pathway in MFT vs. FT, which were identified as *CDPK*, *CAM/CML*, *WRKY*, *PR1*, *BAK1*, *CNGC*, *FLS2* and so on (Fig. S2). Except for *CNGC* and *FLS2*, all of them were up-regulated in MFT vs. FT. Among them, most of *CDPKs* and *CAM/CMLs* were up-regulated in MFT vs. CK, but not differentially expressed in FT vs. CK and MT vs. CK. Differently, *WRKYs* and *BAK1* were down-regulated in FT vs. CK and MT vs. CK, but not differentially expressed in MFT vs. CK. *CNGC* and *FLS2* were down-regulated in MFT vs. CK, but not expressed in FT vs. CK and MT vs. CK (Fig. 5b). It indicated that up-regulation of *CDPKs* and *CAM/CMLs* and down-regulation of *CNGC* and *FLS2* promoted pollen germination in MFT, while down-regulation of *WRKYs* and *BAK1* facilitated incompatibility in FT sample.

#### Response of plant hormone signal transduction to sweetpotato ICI

In plant hormone signal transduction pathway, 27 DEGs were identified in MFT vs. FT, including 19 up- and 8 down-regulated genes. DEGs enriched in auxin, jasmonic acid, brassinosteroid and salicylic acid signaling were all up-regulated in MFT vs. FT, and most of them were enriched in auxin signaling (Fig. 5c), which were identified as *TIR1*, *AUX/IAA*, *GH3*, and *SAUR* (Fig. S2). Expression analysis showed that these identified DEGs were mostly down-regulated or not differentially expressed in FT vs. CK, but conversely expressed in MFT vs. CK (Fig. 5d). It suggested that auxin signaling was an important signal transduction pathway for sweetpotato ICI regulation. DEGs enriched in jasmonic acid, brassinosteroid, and salicylic acid signalings were identified as *BAK1*, *BZR1*, *JAZ*, *TGA*, and *PR1* (Fig. S3). On the contrary, all down-regulated genes were enriched in abscisic acid (ABA), ethylene, and gibberellin signalings in MFT vs. FT (Fig. 5c). Half of them were enriched in ABA signaling and identified as *PYR/PYL*, *PP2C*, and *SnRK2* (Fig. S3), which were all down-regulated in MFT vs. CK, but mostly up-regulated or not differentially expressed in FT vs. CK and MT vs. CK (Fig. 5d). While the DEGs enriched in ethylene signaling were identified as *ETR* and *EBF1* (Fig. S3), which had a similar expression pattern in FT vs. CK and MFT vs. CK (Fig. 5d). Therefore, we speculated that ABA signaling might play a more important role than ethylene signaling in the regulation of sweetpotato incompatibility and promote ICI in FT. DEGs enriched in gibberellin signaling were *GID1* and *DELLA* (Fig. S3). DELLA is the suppressor in gibberellin response. Down-regulation of *DELLA* in MFT vs. CK and MFT vs. FT indicated that gibberellin signaling positively functioned in the regulation of sweetpotato ICI (Fig. 5d).

#### Role of MAPK signaling in the regulation of sweetpotato ICI

MAPK signaling were implicated in flg22, H_2_O_2_, ethylene, ABA, and wounding response (Fig. S4), and the DEGs enriched in flg22, H_2_O_2_, and wounding response, such as *CAM4*, *WRKYs*, *PR1s*, *BAK1*, and *FLS2*, were also the enriched DEGs in plant–pathogen interaction in MFT vs. FT (Fig. S2). The DEGs enriched in ethylene and ABA signaling were corresponding to the plant hormone signal (Fig. S3). Meanwhile, *PR1s* and *BAK1* were also the enriched DEGs in brassinosteroid and salicylic acid signaling. It recommended that MAPK signaling might play a role in the regulation of sweetpotato ICI by linking up the oxidation–reduction, plant–pathogen interaction, and plant hormone signal transduction pathway.

#### Identification of DEGs enriched in pentose and glucuronate interconversions

Pentose and glucuronate interconversions were also the significant enriched pathway in MFT vs. FT. A total of 14 DEGs were enriched in pentose and glucuronate interconversions pathway. Strikingly, the enriched DEGs were mostly identified as *PGs*, *PMEs* and so on, which were also DEGs identified in cell wall metabolism GO terms (Table 2). It indicated that pentose and glucuronate interconversions were important for cell wall mentalism, further demonstrated that cell wall mentalism was important for the regulation of sweetpotato ICI.

**Table 2:** DEGs enriched in pentose and glucuronate interconversions pathway in MFT vs. FT

Gene ID	Log_2_ fold change				Blast Swiss Prot
	FT vs. CK	MT vs. CK	MFT vs. CK	MFT vs. FT	
Tai6.4402	0.4867	0.4775		− 0.4364	Xylose isomerase
Tai6.5918	− 0.2644		0.5422	0.7989	UDP-glucose 6-dehydrogenase 3
Tai6.54227	− 0.2547		0.4954	0.7425	UDP-glucose 6-dehydrogenase 1
Novel00622	− 0.0582			0.1108	Polygalacturonase-like
Novel01078	2.5576		3.2739	0.7088	Polygalacturonase-like
Novel00053	2.1855		3.6773	1.4838	Polygalacturonase-like
Novel00413		− 0.4909	0.6830	0.8644	Polygalacturonase
Novel04207		− 0.8244	0.6358	1.0029	Polygalacturonase
Novel03825	3.1333		3.7079	0.5671	Pectinesterase-like
Novel01307	− 2.6570	− 2.6498		2.2196	Pectinesterase-like
Novel00812	2.5179		3.3405	0.8148	Pectinesterase
Tai6.50313		− 0.1182	− 0.1121	− 0.1493	Pectinesterase
Tai6.22240	− 4.3266	− 2.4114		4.7303	Pectinesterase
Novel02772	2.8223		3.5902	0.7605	Pectinesterase

### Validation of RNA-Seq data and expression of selected DEGs by RT-qPCR

**Fig. 6:**
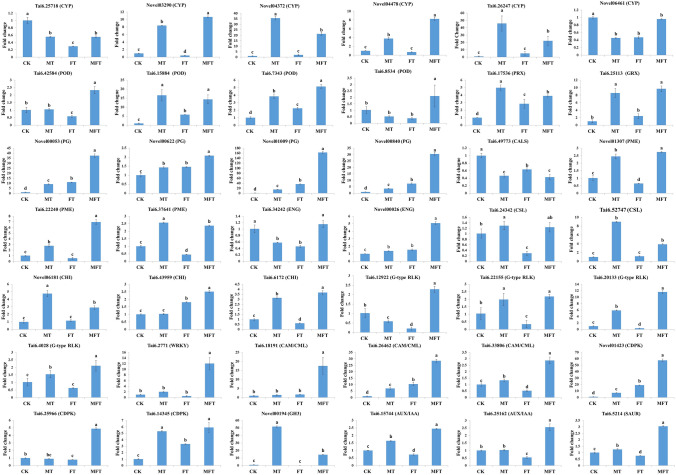
Validation of the expression patterns of the selected DEGs by RT-qPCR. Error bar shows the SD of triplicate assays. Different lowercase letters indicate the significant difference among samples (P < 0.05)

To assess the reliability of RNA-Seq data and validate the expression of the selected DEGs in this study. Forty two genes, including *CYP*, *POD*, *PRX*, *GRX* from oxidation–reduction, *PG*, *PME*, *ENG*, *CSL*, *CHI*, *CALS* from cell wall metabolism, *CAM/CML*, *CDPK*, WRKY from plant–pathogen interaction, *GH3*, *AUX/IAA*, *SAUR* from plant hormone signal transduction, and *G-type RLK* from pollen–pistil interaction, were chosen for the expression analysis by RT-qPCR. The results showed the expression patterns of the 42 DEGs in CK, FT, MT, and MFT were similar between RT-qPCR and RNA-Seq analyses, especially for the expression patterns in FT and MFT compared to CK (Fig. 6). It declared that the RNA-Seq data were credible, which could be used for subsequent experimental analysis, and suggested the selected DEGs in this study were the important candidate genes for sweetpotato ICI regulation.

### Metabonome analysis and identification of DMs for sweetpotato ICI

**Fig. 7:**
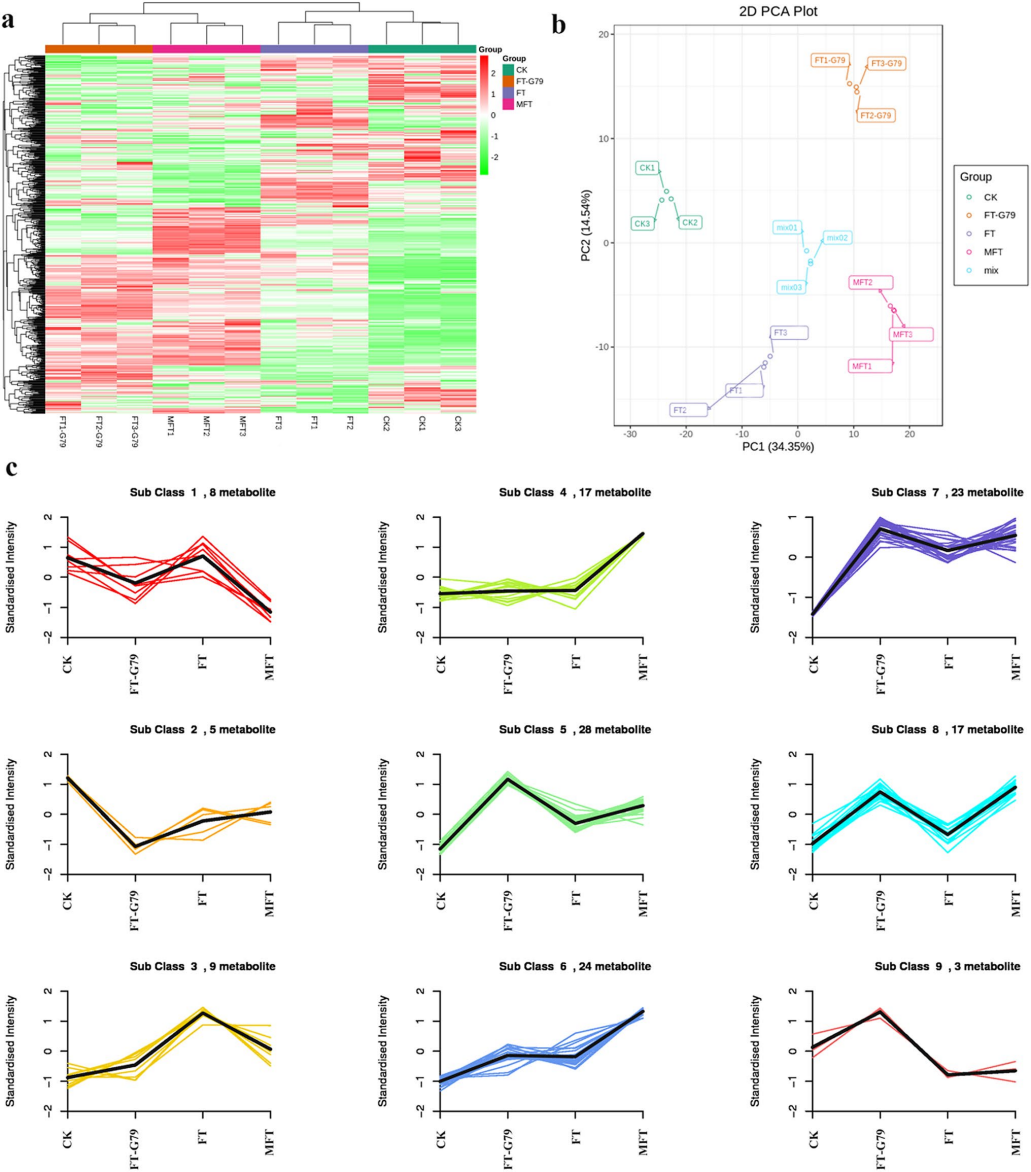
Analysis of metabolites from CK, FT-G79, FT and MFT by hierarchical cluster analysis (HCA), principal component analysis (PCA) and Kmeans_cluster analysis. **a** Analysis of metabolites by HCA. **b** Analysis of metabolites by PCA. **c** analysis of differential metabolites (DMs) by Kmeans_cluster

In this study, metabolome was also employed to further analyze sweetpotato ICI. A total of 612 metabolites were detected in CK, FT, MFT and FT-G79 samples. HCA based on the detected metabolites showed that CK and FT were classed into one group, while FT-G79 and MFT were classed into the other group (Fig. 7a). The result of PCA was consistent with HCA, CK and FT were less than 0, while FT-G79 and MFT were greater than 0 in PC1 (Fig. 7b). It further demonstrated that MFT could promote ICI breaking for sweetpotato.

**Fig. 8:**
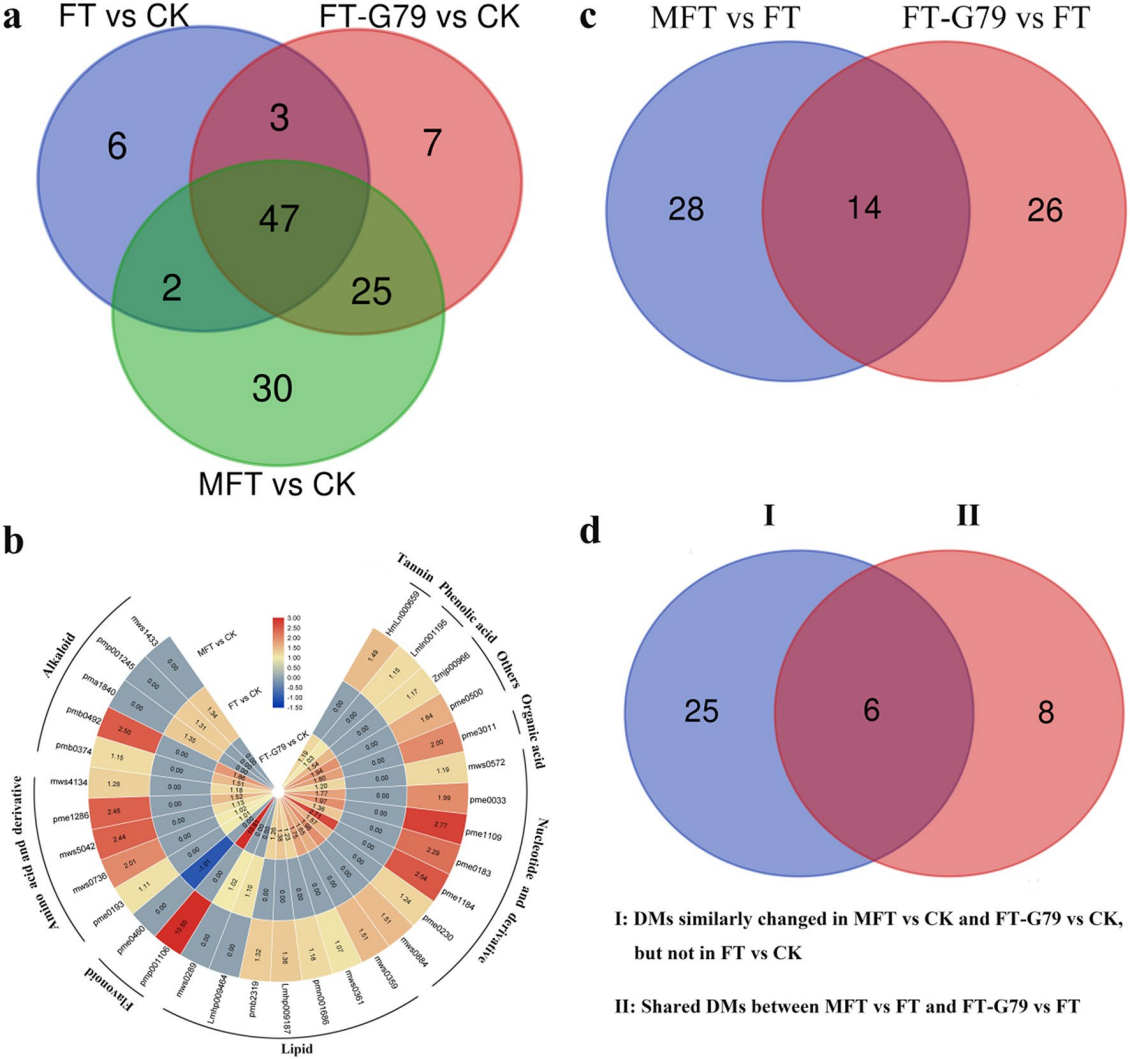
Analysis of DMs in different comparison groups. **a** Venn diagram for DMs from FT-G79 vs. CK, FT vs. CK and MFT vs. CK. **b** Heatmap of DMs similarly changed in FT-G79 vs. CK and MFT vs. CK, but not in FT vs. CK. **c** Venn diagram for DMs from FT-G79 vs. FT and MFT vs. FT. **d** Venn diagram for shared DMs between FT-G79 vs. FT and MFT vs. FT and DMs similarly changed in FT-G79 vs. CK and MFT vs. CK, but not in FT vs. CK

Kmeans_cluster based on the change of metabolite divided all DMs into nine groups. Metabolites from CK and FT performed similar change in groups 1, 5, and 8, but different from FT-G79 and MFT (Fig. 7c), which were identified as alkaloids, lipids, flavonoids, organic acids, amino acids and derivatives, nucleotides and derivatives, tannins, phenolic acids, and others (Table S2). Analysis of DMs in different comparisons showed that there were 120 DMs in FT-G79 vs. CK, FT vs. CK, MFT vs. CK, with 82, 58 and 104 DMs, respectively (Fig. 8a). Among them, 31 DMs performed similar changes in FT-G79 vs. CK and MFT vs. CK, but different from FT vs. CK, including 7 lipids, 5 nucleotides and derivatives, 5 alkaloids, 5 amino acids and derivatives (including a l-glutamine and oxidized glutathione), 2 flavonoids, 1 tannin, 1 phenolic acid, 1 organic acid, and 2 others (Fig. 8b). Among them, 25 DMs were up-regulated in FT-G79 vs. CK and MFT vs. CK, but no difference in FT vs. CK. However, only six DMs showed no difference in FT-G79 vs. CK and MFT vs. CK, but they were up- or down-regulated in FT vs. CK. It further demonstrated that alkaloids, phenolic acids, tannins, lipids, flavonoids, nucleotides and derivatives, amino acids and derivatives, and others might play an important role in sweetpotato incompatibility regulation, and most of them positively promoted ICI breaking for sweetpotato.

Further analysis of DMs from FT-G79 vs. FT and MFT vs. FT showed that there were 42 DMs in MFT vs. FT and 40 DMs in FT-G79 vs. FT, and 14 of them were the shared DMs between the two comparisons and presented consistent change pattern (Fig. 8c). In the 14 shared DMs, 6 of them also performed similar change, being up-regulated, in FT-G79 vs. CK and MFT vs. CK, but showed no different change in FT vs. CK (Fig. 8d), which were identified as vitexin-2-*O*-glucoside (flavonoid), *N*′,*N*″,*N*‴-*p*-Coumaroyl-cinnamoyl-caffeoyl spermidine (Alkaloid), 3-*O*-Galloyl-glucose (Tannin), and three nucleotides and derivatives (Table 3). Vitexin-2-*O*-glucoside was the most significant DM in FT-G79 vs. FT and MFT vs. FT, with log_2_ value being 10.81 and 10.50, respectively. It implied that flavonoids were the important DMs for the regulation of sweetpotato incompatibility. Interestingly, transcriptomic analysis showed flavonoid biosynthesis was also the enriched pathway in MFT vs. FT. *CYP* was the enriched DEG in flavonoid biosynthesis pathway and up-regulated in MFT vs. FT (Fig. S5). It corresponded to the analysis of oxidation–reduction in transcriptome and further supported the function of oxidation–reduction in the regulation of sweetpotato ICI.

**Table 3:** Differentially metabolites shared in FT-G79 vs. FT and MFT vs. FT and performed similar change in MFT vs. CK and FT-G79 vs. CK, but different from FT vs. CK

Index	Compounds	Class	Log_2_ fold change				
			FT-G79 vs. CK	FT vs. CK	MFT vs. CK	FT-G79 vs. FT	MFT vs. FT
pme0033	Hypoxanthine	Nucleotides and derivatives	1.7669	0.0000	1.9856	1.3080	1.5267
pme1109	Guanine	Nucleotides and derivatives	1.9668	0.0000	2.7749	1.0841	1.8922
pme1184	Deoxyguanosine*	Nucleotides and derivatives	2.7105	0.0000	2.5389	1.8904	1.7188
HmLn000659	3-*O*-Galloyl-glucose*	Tannins	1.1886	0.0000	1.4926	1.6209	1.9249
pmb0492	*N*′,*N*″,*N*‴'-*p*-Coumaroyl-cinnamoyl-caffeoyl spermidine	Alkaloids	1.8565	0.0000	2.5030	1.2196	1.8661
pmp001106	Vitexin-2-O-glucoside	Flavonoids	10.8081	0.0000	10.4983	10.8081	10.4983

### ROS detection in incompatibility and compatibility samples.

**Fig. 9:**
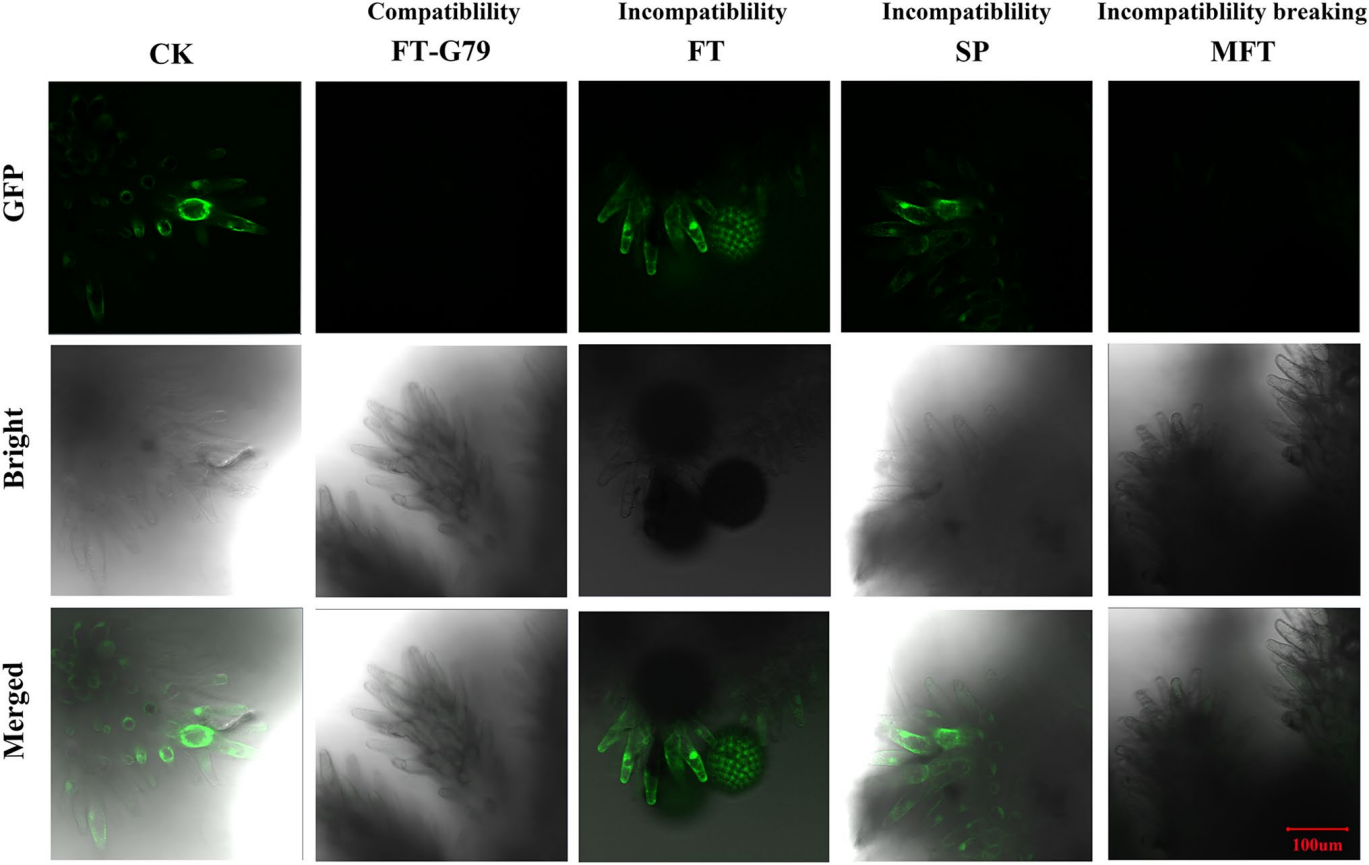
ROS detection in compatible and incompatible samples under laser scanning confocal microscope, with 2′,7′-dichlorodihydrofluorescein diacetate (H2DCFDA) as probe. Scale bar = 100 μm

The results from transcriptome and metabonome analysis agree that oxidation–reduction plays an important role in sweetpotato ICI regulation. To validate the function of the oxidation–reduction, ROS were detected in stigmas of CK, SP, FT-G79, FT and MFT samples under laser scanning confocal microscope, with H2DCFDA as probe. The results showed that the green fluorescence was observed in stigmas of CK, SP and FT, but not in FT-G79, MFT samples (Fig. 9). It further indicated that ROS regulated by oxidation–reduction is the important regulated pathway for sweetpotato ICI.

## Discussion

ICI has been a limiting factor for sweetpotato cross breeding, which is commonly characterized by no pollen germination after cross pollination. Pollen germination detection showed that ‘Guangshu 146’ and ‘Shangshu 19’ were the typical ICI combination, performing no pollen germination in reciprocal cross. However, treatment by inducible reagent screened by Zhang et al. (1998) effectively promoted pollen germination for them. Based on the differential phenotypes between treated and untreated materials, molecular mechanism of sweetpotato ICI was studied by transcriptome and metabolome analysis in this study.

### Screening and analysis of DEGs implicated in sweepotato ICI regualtion by transcriptome

Transcriptome analysis showed that oxidation–reduction was the important pathway for sweetpotato ICI response. ROS is the product regulated by oxidation–reduction. In SI response, ROS has been recognized as a key regulator of programmed cell death, which plays a critical role in rejection of self-incompatible pollen (Wilkins et al. 2011; Serrano et al. 2012, 2015). Recently, ROS produced in stigma papillae has been demonstrated to be a switching factor for the regulation of pollen hydration and germination in *Arabidopsis* (Liu et al. 2021). Similarly, in *B. rapa*, it has been reported that the increase of stigmatic ROS can promote SI, while its decline can break down SI (Zhang et al. 2021b). In this study, 443 DEGs were identified to be involved in oxidation–reduction in MFT vs. FT by GO analysis, with 323 up-regulated genes. Most of them were down-regulated in FT vs. CK and MT vs. CK, while more of them were up-regulated in MFT vs. CK. Analysis of the DEGs showed that multiple DEGs with log_2_ > 1 were identified as *CYP*, *POD*, *PPO*, and *ASO* in MFT vs. FT, especially for *CYP* and *POD*. Most of them were down-regulated in FT vs. CK and MT vs. CK, but not differentially expressed in MFT vs. CK. On the contrary, DEGs identified as *PRX* and *GRX* were up-regulated in MFT vs. FT and MFT vs. CK, but no differentially expressed in FT vs. CK and MT vs. CK. CYP has been extensively identified as regulator in oxidative stress responses (Zhang et al. 2018b). The studies in *Spinacia oleracea* (Duan et al. 2017) and *Gossypium hirsutum* (Magwanga et al. 2019) have reported that CYP can regulate the antioxidant enzymes, such as POD, superoxide dismutase, catalase and glutathione, to scavenge ROS, thus enhancing the tolerance to biotic and abiotic stresses. POD, PPO, ASO, PRX and GRX are the important redox enzymes and proteins regulating intracellular ROS in plant (Gill and Tuteja 2010; Mhamdi and Van Breusegem 2018; Waszczak et al. 2018). A previous study has reported that the increase of POD activity is caused by pollination and pollen tube growth (Bredemeijer 1974). Additionally, recent studies in *Camellia sinensis* have also revealed that there are higher POD and PPO activities in cross-pollinated (compatibility) styles than self-pollinated (incompatibility) styles (Neog et al. 2004; Zhang et al. 2016). It suggested that ROS regulation was essential for the regulation of sweetpotato ICI. The down-regulation of redox-related genes might result in ROS increase and lead to incompatibility in FT sample. Inversely, MFT treatment might fine-tune the ROS environment of pollen and stigma, and promote the pollen germination and pollen tube growth.

KEGG analysis showed that plant–pathogen interaction was the most significantly enriched pathway in MFT vs. FT. It might be attributed to the similarity between pollen–stigma and plant–pathogen interaction (Qu et al. 2017). Because of similar physiological and molecular response mechanisms, the SI system is even hypothesized to originate from pathogen defense system (Bian et al. 2018). DEG analysis revealed that multiple DEGs were identified as *CDPKs* and *CAM/CMLs*, being up-regulated in MFT vs. FT. Most of them were up-regulated in MFT vs. CK, but not expressed in FT vs. CK and MT vs. CK. CDPK and CAM/CML are the important sensors for calcium (Ca^2+^) signal transduction (Zhang et al. 2016). Ca^2+^-mediated signal transduction has been reported to be necessary for the normal pollen germination or tube growth (Nie et al. 2019). In *Arabidopsis*, *CAM/CML* is significantly up-regulated during pollen germination and tube growth (Wang et al. 2008). In *Pyrus pyrifolia*, CAM can promote SI pollen tube growth by enhancing Ca^2+^ influx, ROS concentration, and stabilized actin filaments in pollen (Jiang et al. 2014). In *Agapanthus umbellatus*, CDPK induced by Ca^2+^ exhibits a higher kinase activity in the apical region in growing pollen tube (Moutinho et al. 1998). Similarly, the pollen-specific CDPK is required for maize pollen germination and tube growth (Estruch et al. 1994). Therefore, it was supposed that MFT treatment activated Ca^2+^-dependent signalling cascade in pollen and led to up-regulation of *CDPKs* and *CAM/CMLs* to promote pollen germination and tube growth in the MFT sample. CDPK and CAM/CML in Ca^2+^-dependent signalling cascade can mediate NADPH oxidase (Rboh) to produce ROS. Ca^2+^-activated ROS production by pollen-specific Rboh has been proved to be essential for pollen germination and tube growth (Potock et al. 2007; Kaya et al. 2014). It further supported that ROS played an important role in the regulation of sweetpotato ICI, but might have different functions in FT and MFT samples.

The plant hormone signal transduction was another significant enriched pathway identified in MFT vs. FT by KEGG analysis. DEG analysis showed most of the up-regulated genes in this pathway were enriched in auxin signaling in MFT vs. FT, while most of the down-regulated genes were enriched in ABA and ethylene signaling, especially in ABA signaling. In *Theobroma cacao*, it has reported that auxin is significantly increased in compatibly pollinated flowers, while ethylene and ABA exhibit a significant increase in incompatibly pollinated flowers (Baker et al. 1997). In *Nicotiana tabacum*, auxin is significantly increased in stigma and style when pollen germination and tube growth (Baker et al. 1997). It suggested that auxin and ABA were the key plant hormones to regulate sweetpotato ICI. Expression analysis showed that most of DEGs enriched in auxin signaling from MFT vs. FT were down-regulated or not differentially expressed in FT vs. CK, but conversely expressed in MFT vs. CK, while the down-regulated genes enriched in ABA signaling were all down-regulated in MFT vs. CK, but mostly up-regulated or not differentially expressed in FT vs. CK. It indicated that MFT treatment might trigger auxin increasing and ABA decreasing to facilitate pollen germination and tube growth in the MFT sample.

MAPK signaling was presumed to participate in the regulation of sweetpotato ICI by linking oxidation–reduction, plant–pathogen interaction, and plant hormone signal transduction in this study. In *Papaver*, MAPK signaling has been demonstrated to be involved in mediating SI-induced PCD by activating the MAPKs (p56 and PrMPK9-1) in incompatible pollen (Li et al. 2007; Bosch and Franklin-Tong 2008; Chai et al. 2017). Corresponding to it, *MKK3* was down-regulated in MFT vs. FT (Fig. S4). It indicated that MAPK signal might play a negative role in the MFT sample. MAPK cascades are the signaling modules downstream of RLKs (Zhang et al. 2018a). Strikingly, DEGs enriched in pollen–pistil interaction GO term in MFT vs. FT were all identified as *RLKs*, and DEGs with log_2_ > 1 were all identified as *G-type RLKs*, which were all down-regulated in FT vs. CK but not differentially expressed in MFT vs. CK. SRKs in SI in flowering plants are the best-known members of G-type RLKs (Teixeira et al. 2018). In tea plants, G-type RLKs have also been reported to play potential roles during SI process (Ma et al. 2018). Therefore, it suggested that G-type RLKs were the important RLKs for pollen recognition and signal transduction in sweetpotato ICI, and down-regulation of them might promote ICI in FT samples.

GO and KEGG analyses showed that cell wall metabolism was identified as another important pathway in response to sweetpotato ICI in this study. In apricot (Herrera et al. 2018) and cabbage (Xiao et al. 2019), it has been reported that SI can induce cell wall thickening in pollen tube tip and stigma papilla cells to inhibit pollen germination and tube growth by callose accumulation. Similarly, deposition of callose in stigmatic papilla cells has also been considered to be the reason for inhibition of pollen germination in sweetpotato ICI (Ketong and Shuyun 1992; Shuyun and Taiyuan 1992). Correspondingly, two callose synthases, CALSs, were down-regulated in MFT vs. FT, but two degrading enzymes, ENGs, were up-regulated in this study. It indicated that callose metabolism might play a key role in the regulation of sweetpotato ICI. Conversely, cell wall loosening is requisite for pollen germination and tube growth, which involves the release of enzymes, such as PG, PME and pectic lyase (Dearnaley and Daggard 2001). PG has been reported to be implicated in cell elongation and penetration of pollen tube (Dearnaley and Daggard 2001; Xiao et al. 2014). PME has a bidirectional effect for cell wall metabolism of pollen tube, which can not only stiffen cell wall of tube tip to maintain turgor by deesterifying pectin, but also promote the activity of cell wall hydrolases to lead to cell extension and tube growth by decreasing pH from pectin deesterification (Bosch et al. 2005; Mangano et al. 2016). In this study, 21 and 16 DEGs were identified as PG and PME in MFT vs. FT, and 18 and 13 of them were up-regulated, which indicated that PG and PME might positively function in pollen germination and tube growth in the MFT sample. Furthermore, 11 *CHIs* with log_2_ > 1 were identified in cell wall metabolism pathway in MFT vs. FT. Most of them were down-regulated in FT vs. CK and MT vs. CK, but conversely expressed in MFT vs. CK. CHI activity has been detected in petunia flower, which is mainly kept in stigma and significantly increased following anther dehiscence, indicating that CHI can play a specific role in pollen recognition and germination (Leung 1992; Graham and Stickl 1993). Moreover, because of the parallelism between mycorrhizal hyphae growth in the root and compatible pollen tube in the pistil, CHI is proposed to promote compatible pollen tube growth by degrading or generating signal molecules (Rejón et al. 2014). Therefore, CHI might be an important enzyme for the regulation of sweetpotato ICI.

### Metabolites involved in oxidation–reduction is important for sweetpotato ICI regulation

In addition, metabolome was also employed to explore the mechanism of sweetpotato ICI regulation, associated with transcriptomic analysis. Analysis of DMs showed that 31 DMs were similarly changed in FT-G79 vs. CK and MFT vs. CK, but different from FT vs. CK. They were identified as alkaloids, lipids, flavonoids, organic acids, amino acids and derivatives, nucleotides and derivatives, tannins, phenolic acids, and others. Alkaloid, flavonoid, tannin, and phenolic acid have been reported to be the metabolites implicated in oxidation–reduction regulation (Kristinová et al. 2009; Nakabayashi et al. 2014; Zhang et al. 2018c). Besides, l-glutamine and oxidized glutathione were identified in amino acids and derivatives. They are also the antioxidants to scavenge ROS and protect cell from oxidative stress (Ezhilan et al. 2008; Wang et al. 2019). Most of them were up-regulated in FT-G79 vs. CK and MFT vs. CK, but no change in FT vs. CK. Further analysis of DMs from FT-G79 vs. FT and MFT vs. FT showed that vitexin-2-O-glucoside, belonging to flavonoid, was the most significant DM both in FT-G79 vs. FT and MFT vs. FT and also up-regulated in FT-G79 vs. CK and MFT vs. CK, but no differential change in FT vs. CK. It suggested that flavonoid was the important metabolite for the regulation of sweetpotato ICI. Transcriptomic analysis showed that a *CYP* was the enriched DEG in flavonoid biosynthesis pathway in MFT vs. FT, being up-regulated. CYPs have been reported to be involved in the flavonoid metabolism, including the biosynthesis of anthocyanin pigments, condensed tannin, flavone and leguminous isoflavonoid phytoalexins (Ayabe and Akashi 2006). Multiple *CYPs* were also identified in oxidation–reduction pathway in transcriptomic analysis. Taken together, it further illustrated that oxidation–reduction was the important pathway to regulate sweetpotato ICI, which was validated by ROS detection in incompatible and compatible samples.

### Putative response model for regulation of sweetpotato ICI

**Fig. 10:**
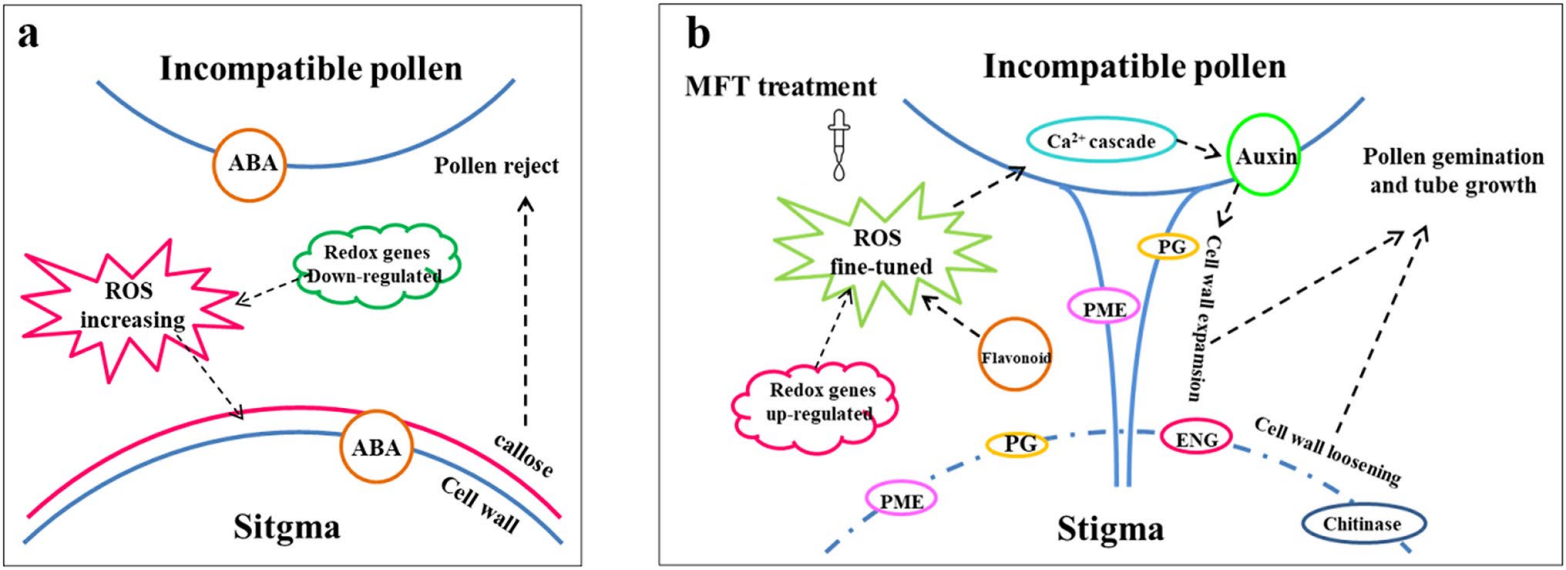
The putative response model for the regulation of sweetpotato ICI. **a** The putative response mechanism for sweetpotato ICI. **b** The putative response mechanism for sweetpotato ICI breaking by MFT treatment

Based on the above-mentioned results, a putative response model for the regulation of sweetpotato ICI was proposed in this study (Fig. 10). Oxidation–reduction was the switch point for the model. When the incompatible pollens landed on the stigma, the redox-related genes, such as *CYP* and *POD*, were more down-regulated, ROS was increased rapidly, ABA signaling was triggered, and callose was subsequently deposited in stigmatic papilla cells to strengthen cell wall, finally resulting in ICI response. On the contrary, in MFT samples, ROS environment was fine-tuned, the redox-related genes, such as *PRX* and *GRX*, were more up-regulated, metabolites, such as flavonoids, were accumulated, ROS was decreased, and Ca^2+^ signalling cascade was activated. Meanwhile, auxin signaling was triggered, and the enzymes, such as ENGs, PGs, PMEs and CHIs, were released to loosen pollen and stigma cell wall to promote pollen germination and tube growth.

## Supplementary Information

Below is the link to the electronic supplementary material.

Supplementary file1 (DOCX 2016 kb)

Supplementary file2 (DOCX 22 kb)

Supplementary file3 (XLSX 14 kb)

## Data Availability

All data and materials support the published claims and comply with field standards. All data generated or analysed during this study are included in this published article and its Supplementary Information files. The transcriptomic sequence data generated during the current study are available in the NCBI SRA Repository under the accession number PRJNA611841, https://www.ncbi.nlm.nih.gov/sra/?term=PRJNA611841.
