# Supplementary material for: Exploration of molecular mechanism of intraspecific cross-incompatibility in sweetpotato by transcriptome and metabolome analysis
Source: Plant Mol Biol. 2022 Mar 25;109(1-2):115–33. doi: 10.1007/s11103-022-01259-8 (PMC9072463; doi:10.1007/s11103-022-01259-8)
Supplement: Supplementary file 1 — Supplementary file1 (DOCX 2016 kb) [file 11103_2022_1259_MOESM1_ESM.docx]

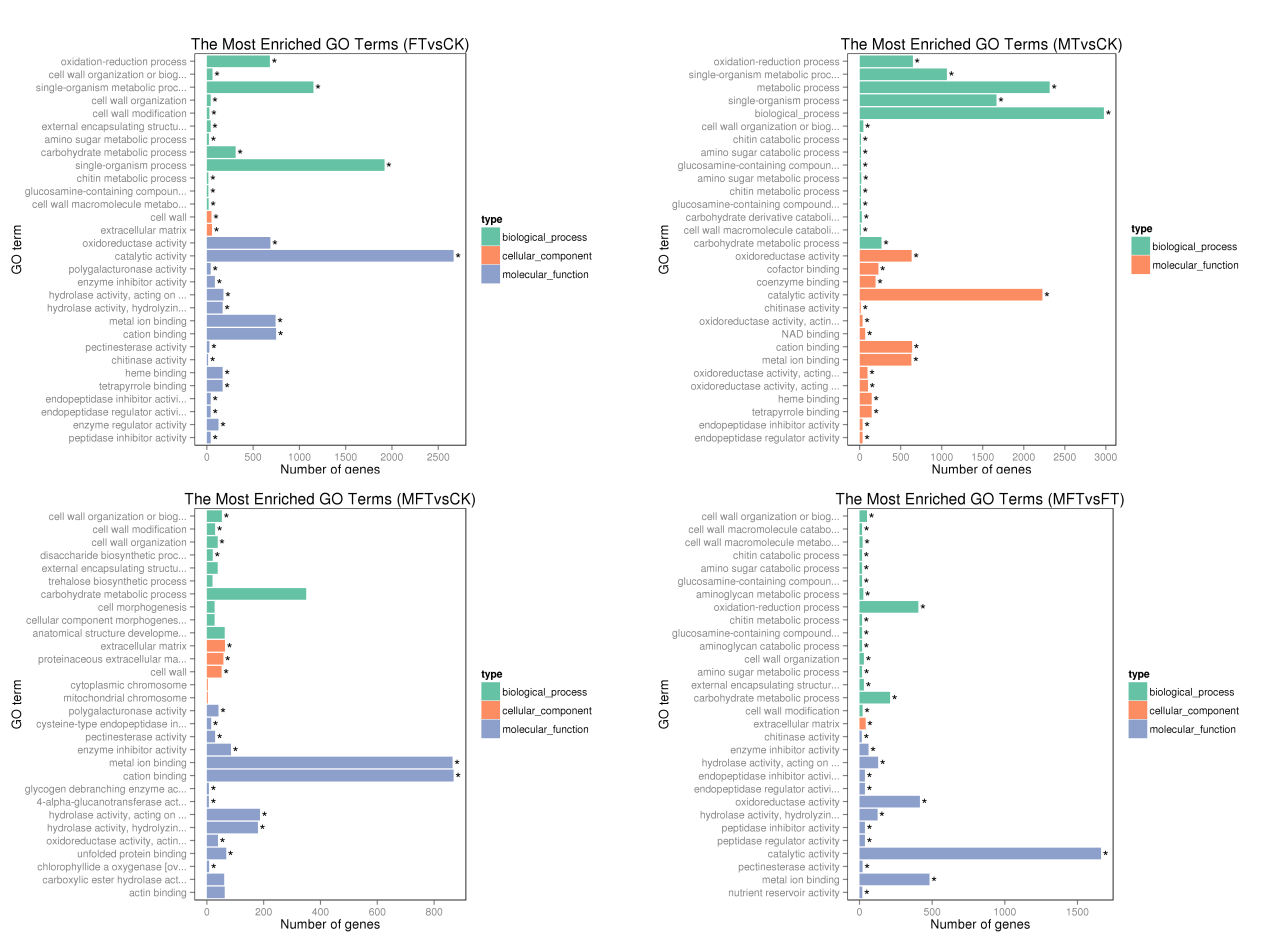


**Fig. S1** The top 30 GO terms in FT vs CK, MT vs CK, MFT vs CK and MFT vs FT.


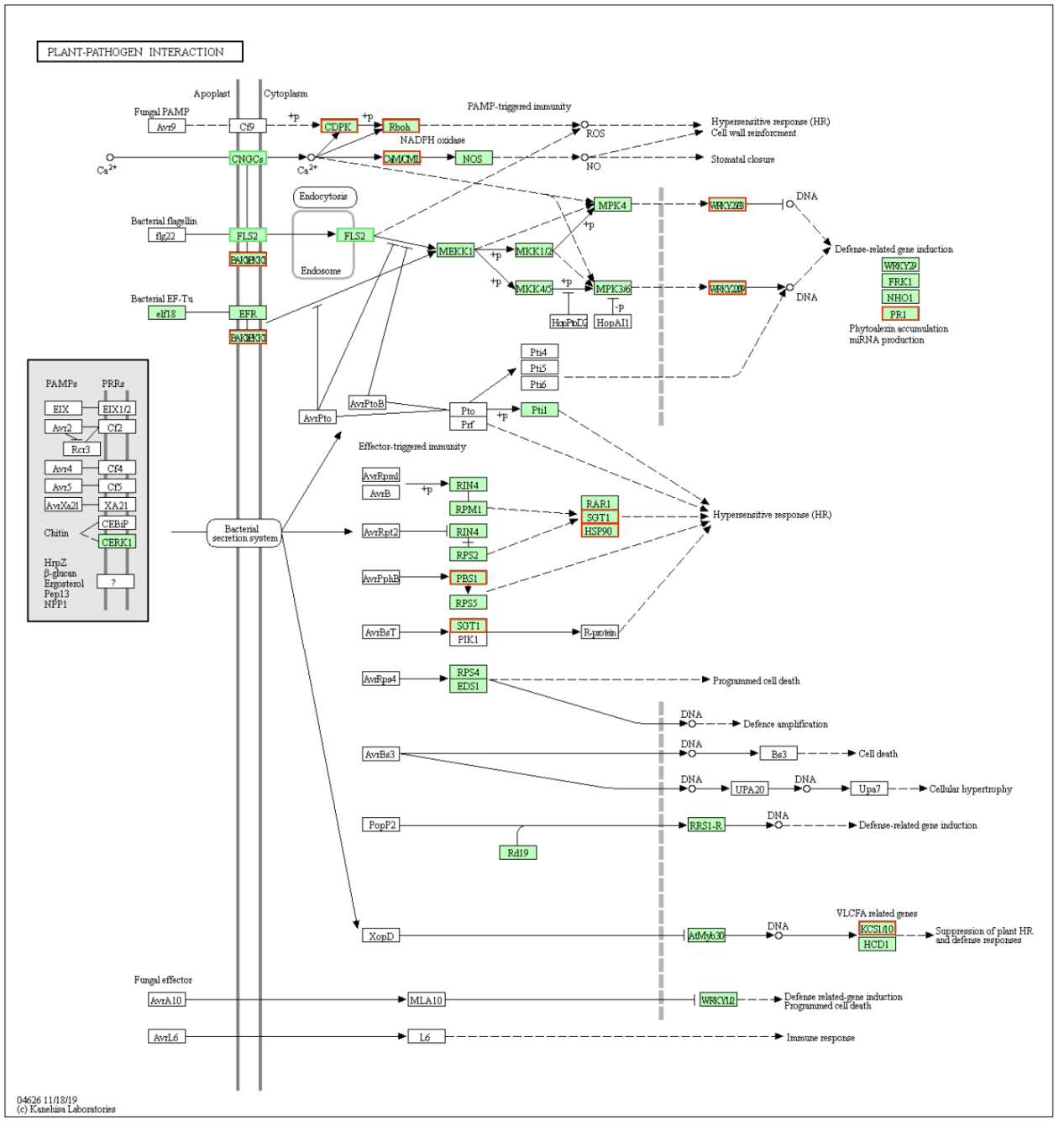


**Fig. S2** Enrichment diagram of plant-pathogen interaction pathway in MFT vs FT. The green frame outlines represent nodes with down-regulated DEGs, red outlines represent nodes with up-regulated DEGs.


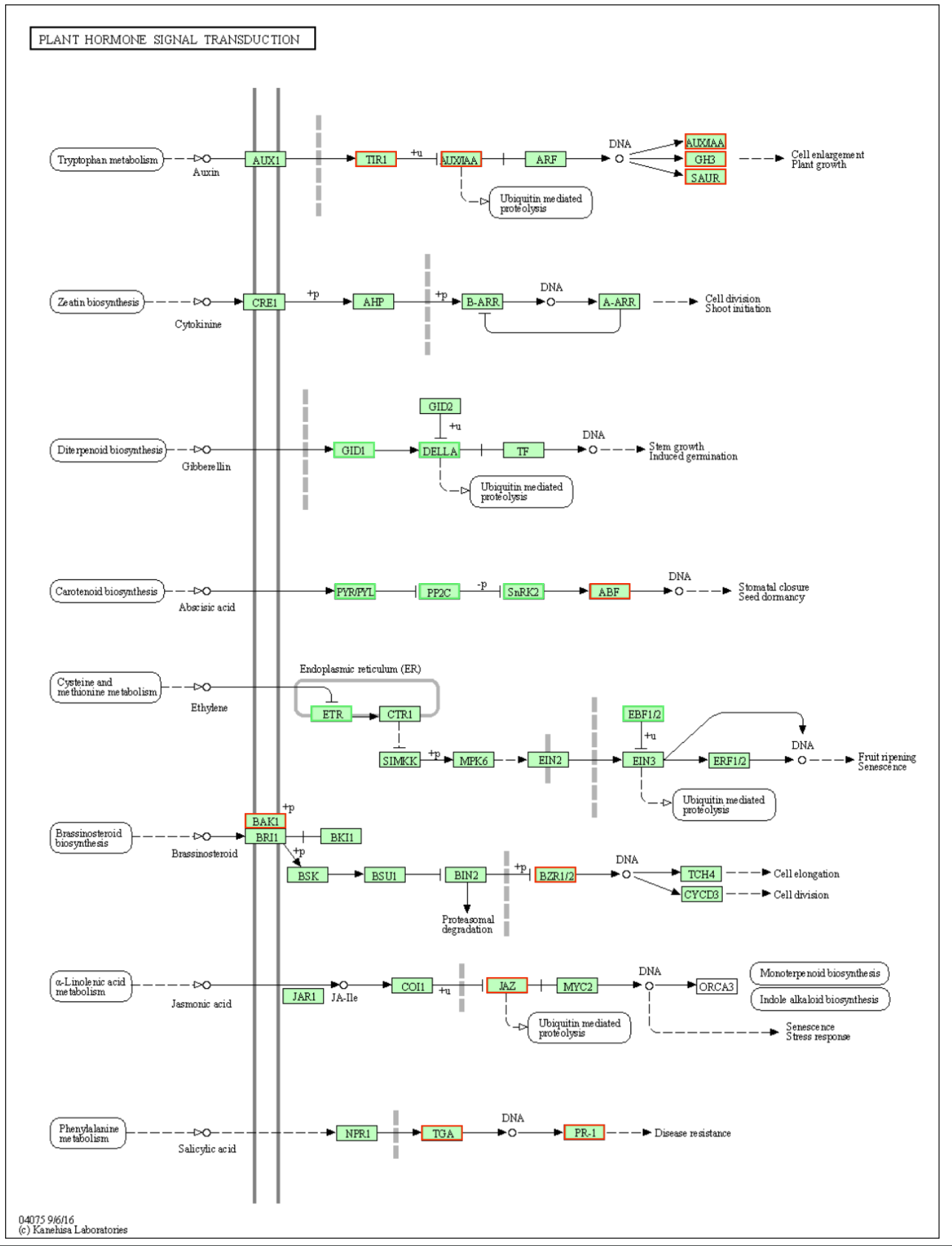


**Fig. S3** Enrichment diagram of plant hormone signal transduction pathway in MFT vs FT. The green frame outlines represent nodes with down-regulated DEGs, red outlines represent nodes with up-regulated DEGs.


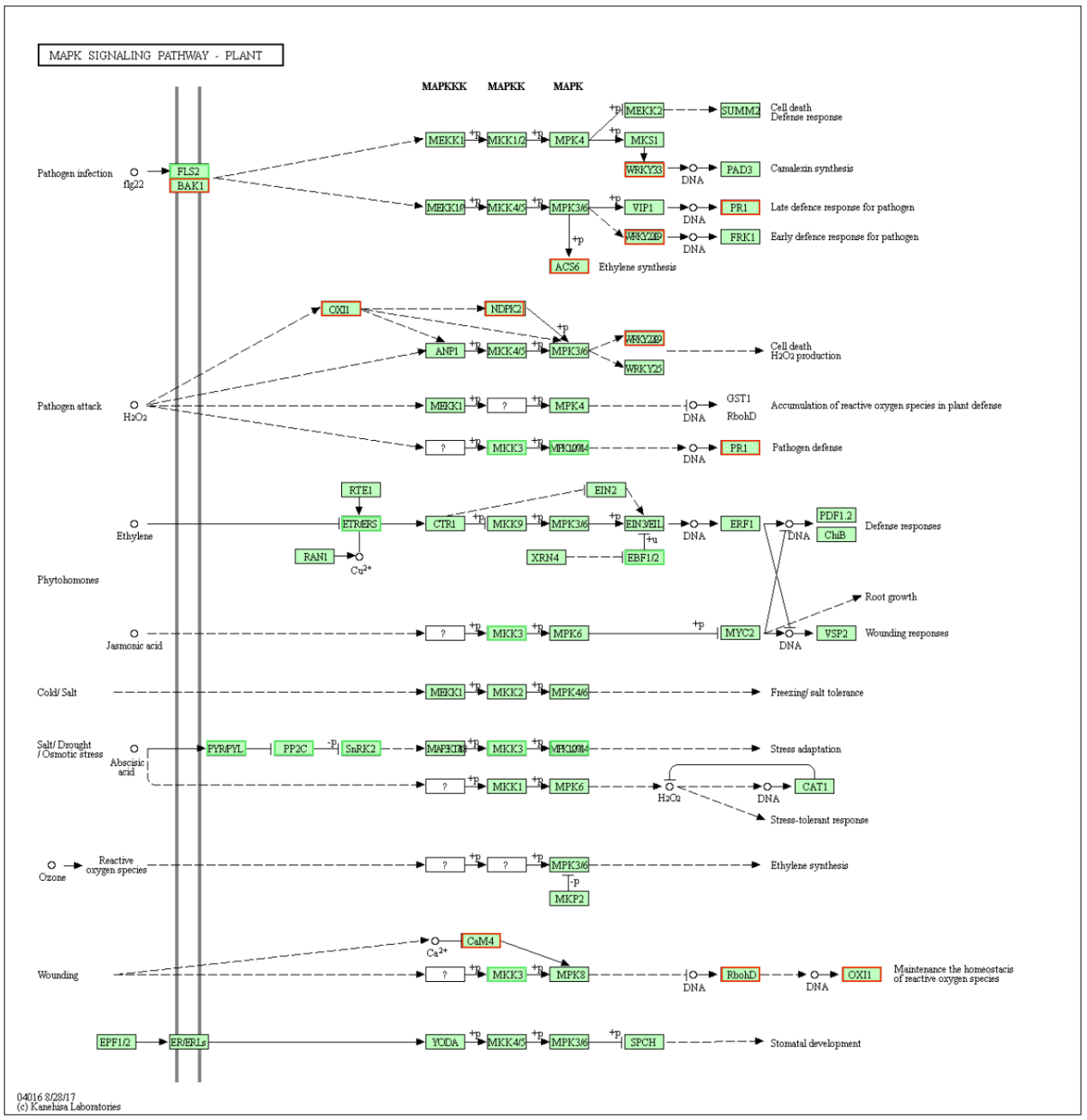


**Fig. S4** Enrichment diagram of MAPK signaling pathway in MFT vs FT. The green frame outlines represent nodes with down-regulated DEGs, red outlines represent nodes with up-regulated DEGs.


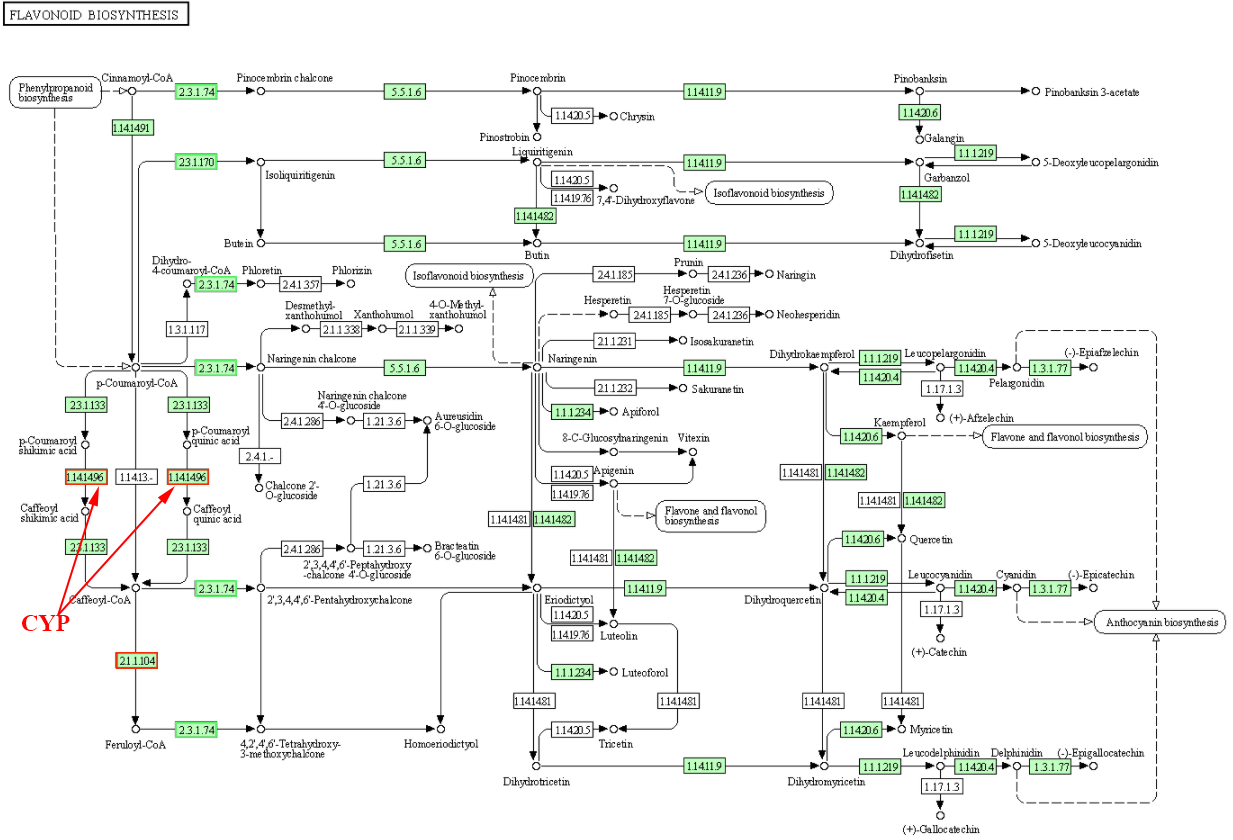


**Fig. S5** Enrichment diagram of flavonoid biosynthesis pathway in MFT vs FT. The green frame outlines represent nodes with down-regulated DEGs, red outlines represent nodes with up-regulated DEGs. The red arrows show the nodes enriched by *CYP*.
