# Supplementary material for: Exploration of molecular mechanism of intraspecific cross-incompatibility in sweetpotato by transcriptome and metabolome analysis
Source: Plant Mol Biol. 2022 Mar 25;109(1-2):115–33. doi: 10.1007/s11103-022-01259-8 (PMC9072463; doi:10.1007/s11103-022-01259-8)
Supplement: Supplementary file 2 — Supplementary file2 (DOCX 22 kb) [file 11103_2022_1259_MOESM2_ESM.docx]

**Table S1** Summary of mapped reads based on the sweetpotato genome

| Sample name | Raw reads | Clean reads | Q20(%) | Total mapped | Multiple mapped | Uniquely mapped |
| --- | --- | --- | --- | --- | --- | --- |
| CK | 163180202 | 156228466 | 96.70 | 118802404  (76.04%) | 7366272  (4.71%) | 111436132  (71.33%) |
| FT | 163492116 | 155806802 | 96.82 | 118424123  (76.01%) | 7459743  (4.79%) | 110964380  (71.22%) |
| MT | 161875494 | 155355808 | 96.60 | 117920364  (75.91%) | 7240022  (4.66%) | 110680342  (71.25%) |
| MFT | 148857706 | 142765672 | 96.67 | 107203849  (75.07%) | 6586841  (4.60%) | 100617008  (70.47%) |
| Total | 637405518 | 610156748 |  | 462350740 |  |  |

**Table S3** Sequences of the primers for qRT-PCR

| Gene name | Primer direction | Primer sequence (5' to 3') |
| --- | --- | --- |
| Actin | Forward | ACTGGAACAGCCAGAGGAGA |
|  | Reverse | ATGCAATCTTCCATGGGTTC |
| Novel06461 | Forward | CAACTAGGGTTGGCCAGTGT |
|  | Reverse | TGGGGTGAGGAGAGCTTCTA |
| Tai6.26247 | Forward | AGGGTGAACGTCAGATTGGG |
|  | Reverse | GGGATGGCTATTGCATTTCCAG |
| Novel04478 | Forward | ACCTTGTTGGAAACCATGGAA |
|  | Reverse | CCGTCGCTGACGTATCCATT |
| Novel04372 | Forward | ACTCAATCGCGACCGCATAA |
|  | Reverse | ATCCGTCGCCTTTTCGATGA |
| Novel03290 | Forward | CAGTTCCCCATCACCATCCAA |
|  | Reverse | TCTACCATTGCCCACACGACC |
| Tai6.25718 | Forward | TCCCAGAGAGGTTTGAAGGGA |
|  | Reverse | ATTCCAAGGTGCAGACCAGG |
| Tai6.8534 | Forward | ACCATTGTGCATAAATCAAGCC |
|  | Reverse | CGCTTCATCGCCAAGCTTAC |
| Tai6.42584 | Forward | AATGGGGATGGGGAGATGGA |
|  | Reverse | TGTGACAAAAGTCTGGGCGA |
| Tai6.7343 | Forward | CTGTCATCACTTCCAATGCACC |
|  | Reverse | ACAGACTCAATTGTGAGGGGAT |
| Tai6.15884 | Forward | CGCTGAGAGGGTTCGACTTT |
|  | Reverse | CGGTGGGGACATTCCAGAAA |
| Tai6.25113 | Forward | ACCACCTTGTCGCTTTAGGG |
|  | Reverse | CCTCCTACATGGAGGCGTTT |
| Tai6.17536 | Forward | CCGTTCCAAGAAAACGGCAA |
|  | Reverse | TAGTTGTGGTGGTGACGTGG |
| Novel00053 | Forward | AGCACGTTTCGGAGAGAGTG |
|  | Reverse | GTCCCACCACCTGTTAGTGT |
| Novel01009 | Forward | TGATGGGTTTAAAACTCCTTTGC |
|  | Reverse | TGCGTCCTTCCAAGCTTTCT |
| Novel00840 | Forward | AGTGTAATCACGGATAGTTAGACAA |
|  | Reverse | GCCAGCATCATATCCTAGCTCA |
| Tai6.24342 | Forward | GTCGATACGAGCATGGTGGA |
|  | Reverse | CCGCTTTTCCCTGCGTAAAT |
| Tai6.52747 | Forward | ATGGGTCCCGTTTTGTCGAA |
|  | Reverse | AACTCATCGTACTCGCGCTT |
| Tai6.22240 | Forward | AACCCAGCCACGTTACTCTG |
|  | Reverse | ACGGATCCCAACCAGAACAC |
| Tai6.37641 | Forward | CATTTTGCCCGCCGTGAC |
|  | Reverse | CTCCCACTTCTCTTAGCCGC |
| Novel01307 | Forward | ACCGGTTTTGGGCTCTTTCA |
|  | Reverse | TAACTCGGTACCCACCCCAT |
| Tai6.34242 | Forward | TACCAGGTTTTCAACGCGGA |
|  | Reverse | GGGGGAAGAGGCGTACAAAA |
| Novel00026 | Forward | CCACGCGGTTCTTCCTGC |
|  | Reverse | TCTTGTGGCAATCGGGTCTA |
| Tai6.49773 | Forward | AGTTTTGTGACACTGGGGCA |
|  | Reverse | CACTAGCGACCTTAGCCTCG |
| Novel06181 | Forward | GAGACTACTGCGACAGGACC |
|  | Reverse | TGCATTCAAGTTGGCCGTTG |
| Tai6.6172 | Forward | CGGTCTAAACAACCCGGACA |
|  | Reverse | TTCGGATTGGCACCATCACA |
| Tai6.43959 | Forward | TGGATGACACCCCAATCCC |
|  | Reverse | ATCAAGATTGCTCCCAACCCC |
| Tai6.12922 | Forward | TGTGGAGGTAGGCCAAGATA |
|  | Reverse | GCCCAGTAAGCATTTCCACC |
| Tai6.4028 | Forward | GGGTGGCTGCTTCAGAGATT |
|  | Reverse | TCATGTTATGCCCTCGCCTC |
| Tai6.20133 | Forward | AGCCTGCAGATTTGTGTGGG |
|  | Reverse | TTCTCGCGTTCCATTGGTTC |
| Tai6.22155 | Forward | CCATCACGGCCAAAGCAAAC |
|  | Reverse | CCTTGGGGTCCAGAAGTCC |
| Tai6.2771 | Forward | ACCAAGCAATCCACCACCATA |
|  | Reverse | TTGACTGGTTTTTGCTGCGT |
| Tai6.33806 | Forward | CCCATTCCCAGATTCCCAGC |
|  | Reverse | ACCAAGGGCGAAGAGAAGTG |
| Tai6.18191 | Forward | GCAGCAAGCTTAGAGCAGC |
|  | Reverse | TGAAGGCCAATGAGCCTAAG |
| Tai6.26462 | Forward | TCATCACTCGCAACCATGGAA |
|  | Reverse | GAACATGTCGAAGATGCGGC |
| Tai6.14345 | Forward | GGTCTGAGAACACTGCCACA |
|  | Reverse | TTTGACTCCTGTGACTGGGC |
| Tai6.25966 | Forward | TTCCCGGGACATGCTTTGTT |
|  | Reverse | TCGGAGATTCGGGTTTTGGG |
| Novel01423 | Forward | GCAACTCATGGAAGCTGCTG |
|  | Reverse | TTTTCCGTCACTCATGCCGA |
| Tai6.25162 | Forward | ATGCATTTCTCAGACGGGCT |
|  | Reverse | CGCTCGGGAGATTGAGATCC |
| Tai6.15744 | Forward | CTCCGGCTAGGGTTGCC |
|  | Reverse | GCTGGTGGCTTAGTGGAGTC |
| Novel00194 | Forward | TCACTTGTCTTATTGCCACCA |
|  | Reverse | GAGAGGGGAAGACGACACAA |
| Tai6.5214 | Forward | GCCAGGCATCCTTTCGGTAT |
|  | Reverse | CGATCCCTGGCCTTTCTCTC |
| Novel00622 | Forward | TAACATGTACACTAGCTGCCAAT |
|  | Reverse | ATGGGGCCACAATTGTTCCAT |
| Novel00413 | Forward | CGGCAACATTCTACCTTGTCC |
|  | Reverse | ATCCCTTTGCATGGAACCGT |
